# Small RNA and transcriptome deep sequencing proffers insight into floral gene regulation in *Rosa *cultivars

**DOI:** 10.1186/1471-2164-13-657

**Published:** 2012-11-21

**Authors:** Jungeun Kim, June Hyun Park, Chan Ju Lim, Jae Yun Lim, Jee-Youn Ryu, Bong-Woo Lee, Jae-Pil Choi, Woong Bom Kim, Ha Yeon Lee, Yourim Choi, Donghyun Kim, Cheol-Goo Hur, Sukweon Kim, Yoo-Sun Noh, Chanseok Shin, Suk-Yoon Kwon

**Affiliations:** 1 Green Bio Research Center, 125 Gwahak-ro, Yuseong-gu, Daejeon, 305-806, Republic of Korea; 2 Bioinformatics, University of Science & Technology, 217 Gajung-ro, Yuseong-gu, Daejeon, 305-350, Republic of Korea; 3 School of Biological Sciences, Seoul National University, 1 Gwanak-ro, Gwanak-gu, Seoul, 151-747, Republic of Korea; 4 Korea Ocean Research & Development Institute, 787 Haeanro, Sangrok-gu, Ansan, 426-744, Republic of Korea; 5 Biological Resource Center, Korea Research Institute of Bioscience and Biotechnology, 125 Gwahak-ro, Yuseong-gu, Daejeon, 305-806, Republic of Korea; 6 Biosystems and Bioengineering Program, University of Science & Technology, 217 Gajungno, Yuseong-gu, Daejeon, 305-350, Republic of Korea; 7 Department of Agricultural Biotechnology, Seoul National University, 1 Gwanak-ro, Gwanak-gu, Seoul 151-921, Republic of Korea

## Abstract

**Background:**

Roses (*Rosa* sp.), which belong to the family Rosaceae, are the most economically important ornamental plants—making up 30% of the floriculture market. However, given high demand for roses, rose breeding programs are limited in molecular resources which can greatly enhance and speed breeding efforts. A better understanding of important genes that contribute to important floral development and desired phenotypes will lead to improved rose cultivars. For this study, we analyzed rose miRNAs and the rose flower transcriptome in order to generate a database to expound upon current knowledge regarding regulation of important floral characteristics. A rose genetic database will enable comprehensive analysis of gene expression and regulation via miRNA among different *Rosa* cultivars.

**Results:**

We produced more than 0.5 million reads from expressed sequences, totalling more than 110 million bp. From these, we generated 35,657, 31,434, 34,725, and 39,722 flower unigenes from *Rosa hybrid*: ‘Vital’, ‘Maroussia’, and ‘Sympathy’ and *Rosa rugosa* Thunb. , respectively. The unigenes were assigned functional annotations, domains, metabolic pathways, Gene Ontology (GO) terms, Plant Ontology (PO) terms, and MIPS Functional Catalogue (FunCat) terms. Rose flower transcripts were compared with genes from whole genome sequences of Rosaceae members (apple, strawberry, and peach) and grape. We also produced approximately 40 million small RNA reads from flower tissue for *Rosa*, representing 267 unique miRNA tags. Among identified miRNAs, 25 of them were novel and 242 of them were conserved miRNAs. Statistical analyses of miRNA profiles revealed both shared and species-specific miRNAs, which presumably effect flower development and phenotypes.

**Conclusions:**

In this study, we constructed a Rose miRNA and transcriptome database, and we analyzed the miRNAs and transcriptome generated from the flower tissues of four *Rosa* cultivars. The database provides a comprehensive genetic resource which can be used to better understand rose flower development and to identify candidate genes for important phenotypes.

## Background

Roses (*Rosa* sp.) belong to the Rosaceae family and are the most important ornamental plants, comprising 30% of the floriculture market. The Rosaceae family consists of more than 100 genera and 3,000 species, including many important fruits, nuts, ornamental, and wood crops [1]. Members of this family provide high-value nutritional food and contribute desirable aesthetic and industrial products. In addition, there are abundant genomic resources from recently released genome sequences for apple, strawberry, and peach (http://www.rosaceae.org/) that will contribute to better understanding of Rosaceae biology [2,3]. Despite active genomic studies of fruit-bearing Rosaceae, molecular studies of ornamental roses have been limited, except for those focused on supporting breeding strategies.

The development of molecular markers for roses began with the first molecular linkage map covering over 300 markers from *Rosa multiflora* hybrids [4], and several genetic maps were constructed recently [5,6]. However, the genetic resources for roses are relatively weak compared to those for other Rosaceae families. As of June 2011, approximately 4,834 unigenes were available. These were generated from 9,289 expressed sequence tags (ESTs) for rose in the Genome Database for Rosaceae (GDR) [7,8]. These unigenes cover only 7.6% of apple genes, 13.89% of strawberry genes, and 16.84% of peach genes. Clearly, more abundant transcriptomic resources generated from different roses are needed to allow for the investigation of key traits, including resistance to disease or stress, flower morphology, and scent [9,10].

Transcriptome sequences are often analyzed from both model and non-model plants to monitor whole gene expression. Whole gene expression is useful to identify biotic [11] or abiotic stress related genes [12,13], understand organ development [14,15] and characterize differential traits between closely related species of rose [16]. Next-generation sequencing (NGS) technologies, such as Illumina, SOLEXA, Genome Sequencer FLX system (GS FLX), and ABI SOLiD, allow analysis of the transcriptome because of increased throughput and reduced sequencing cost [17,18]. The GS FLX is considered by many to be the most powerful platform to analyze protein-coding sequence data with strengths, including long reads, good accuracy, and the ability to support ultra-high-throughput analysis [19]. Because of these strengths, GS FLX is often applied to generate transcriptome data (summarized in Table 1 in [20]). Transcriptomic data in ornamental plants like roses expands our knowledge of the genetic control of flower quality. These findings can be applied in the floricultural industry to advance efforts to screen economically important phenotypes [21]. Here, we generated transcriptomes of four *Rosa* cultivars using GS FLX sequencing to compare floral development and other features.

**Table 1:** Summary of sequencing and assembly results for rose flowers

	**V**	**M**	**S**	**H**
**Raw data**				
Read No.	151,906	132,974	107,816	115,707
Read length (bp)	34,317,306	29,145,021	23,643,473	26,107,242
Avg. read length (bp)	225.91	219.18	219.30	225.63
**Statistics after pre processing**				
Read No.	142,421	116,397	95,875	109,067
	(93.76 %)	(87.53 %)	(88.92 %)	(94.26 %)
Read length (bp)	33,112,590	27,277,537	22,306,881	25,260,565
Avg. read length (bp)	232.50	234.35	232.67	231.61
**Sequence clustering and assembly**				
Clustered reads	120,931	97,250	73,768	84,281
Clustered range	2-4,792	2-3,726	2-1,353	2-980
Contigs (assembled by TGICL ^a^)	14,167	12,287	12,618	15,366
Singlets ^b^	3,501	3,261	3,406	3,814
Singlets ^c^	17,989	15,886	18,701	20,542
Avg. contigs length (bp)	397.60	441.52	416.45	369.33

^a ^TGICL: TIGR Gene Index CLustering tools, ^b ^reads that are clustered but not assembled into contigs, ^c ^un-clustered reads by TGICL.

Abbreviations: V: ‘Vital’; M: ‘Maroussia’; S: ‘Sympathy’; H: ‘Haedang’.

MicroRNAs (miRNAs) are short (20–24 nt) non-protein-coding RNAs [22,23], which play essential roles in regulating plant growth and development [22]. miRNA genes, called *MIR* genes, are transcribed by RNA polymerase II (Pol II), and the miRNA transcripts form self-complementary fold-back structures called primary miRNA (pri-miRNA). Pri-miRNAs are processed to mature RNAs (miRNA/miRNA* duplex) through cleavage by Dicer-like 1 (DCL1) protein [23]. After release into the cytosol, miRNAs bind near-perfectly to their target mRNAs, and the remaining strands (miRNA*) are degraded. miRNAs regulate expression of target mRNA post-transcriptionally through either cleavage of the target mRNA or translational inhibition.

miRNAs are often identified by cloning or by bioinformatic alignment to known miRNAs. However, several strategies have been developed to computationally identify miRNAs from deep sequencing data [24-27]. These algorithms, which were initially designed for vertebrate miRNAs, are now able to predict plant miRNAs considering different features of plant miRNAs [28,29]. There are several databases of annotated and predicted miRNAs for plants that can be used in bioinformatic approaches for miRNA identification, including miRBase [30] and the Plant miRNA Database (PMRD) [31]. These recent developments of high-throughput sequencing techniques and analysis tools, as well as databases for miRNAs, challenge us to identify miRNAs from various plant species.

In this study, we constructed a Rose database of transcriptomes and miRNA sequences and annotations generated from flower tissues of three *Rosa hybrid* cultivars (Vital, Maroussia, and Sympathy) and *R. rugosa* Thunb. (Haedang). We included data from the three sequenced Rosaceae genomes (apple, strawberry, and peach) and the grape genome. In the rose database, users can analyze gene contents, gene families, phylogenetics, and miRNA profiles from a publicly available website (http://210.218.199.249/rose/). These data can be used to identify candidate miRNAs and target genes that may regulate flower development and hormonal regulation. It can be used to establish a genetic basis of valuable phenotypes of rose flowers. To demonstrate the utility of the database, we describe miRNAs that are highly conserved among *Rosa* (‘Vital’, ‘Maroussia’, ‘Sympathy’, and ‘Haedang’), as well as statistically abundant miRNAs in one rose than others. Several target genes of miRNAs were experimentally validated using 5' RACE, and most of them were transcription factors regulating flower development. Therefore, rose database provides a comprehensive resource to understand flower development and to identify new candidate genes for studying rose phenotypes.

## Results

### Transcriptome sequence assembly from rose flower libraries

**Figure 1:**
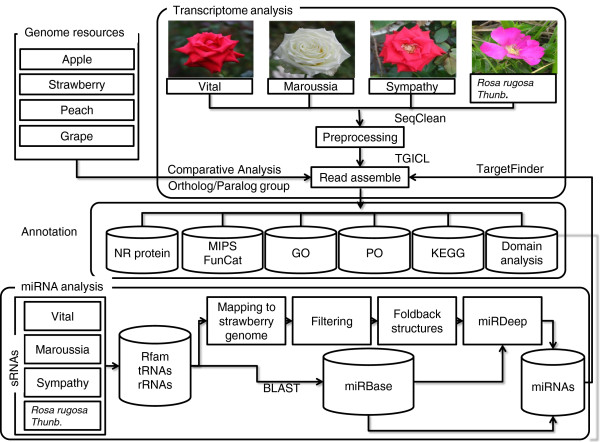
**Scheme for construction of the Rose database. **The schematic presentation shows the strategy of transcriptome and miRNA analysis. Transcript reads were assembled by TGICL [32] after pre-processing with SeqClean. Assembled sequences were annotated by searching homologs against non-redundant (NR) database and Arabidopsis (ver. 10). Domains and Gene Ontology (GO) were analyzed by Pfam. KEGG metabolic pathways and plant ontology (PO) were assigned by referring the description of top matched *Arabidopsis *genes. miRNAs were identified by applying modified miRDeep [24,29]. To search for known miRNAs, sRNA sequences were BLAST searched against currently known miRNAs in miRBase [86] databases.

We constructed sequencing libraries from complete flower tissue of three *Rosa hybrid* cultivars, including Red corvette (Vital), Maroussia, and Sympathy, and *R. rugosa* Thunb. (Haedang), and sequenced them using the GS FLX platform. A total of 151,906 (approximately 34.3 Mb), 132,974 (29.1 Mb), 107,816 (23.6 Mb) and 115,707 (26.1 Mb) reads were generated from ‘Vital’, ‘Maroussia’, ‘Sympathy’, and ‘Haedang’, respectively (Table 1). The average read length was 219–226 bp for each library. Prior to assembly, we masked low-complexity and poly (A/T) sequences and removed reads shorter than 100 bp using the SeqClean program downloaded from the Dana Farber Cancer Institute (http://compbio.dfci.harvard.edu/tgi/software/) (Figure 1). After pre-processing, 88-94% of raw data remained to be clustered and assembled into contigs (Table 1).

To construct contigs, the TIGR Gene Index CLustering tools (TGICL) package implements MGBLAST to collect reads into clusters, and Cap3 was used to assemble clustered reads into contigs [32]. After clustering, clusters contained 84.91%, 83.55%, 76.94%, and 77.27% of the pre-processed reads for ‘Vital’, ‘Maroussia’, ‘Sympathy’, and ‘Haedang’, respectively (Table 1). Using the clusters, the cap3 constructed 14,167, 12,287, 12,618, and 15,366 contigs for ‘Vital’, ‘Maroussia’, ‘Sympathy’, and ‘Haedang’, respectively. Those contigs contained 95-97% of the clustered reads. Average contig lengths were 397.60, 441.52, 416.45, and 369.33 bp for ‘Vital’, ‘Maroussia’, ‘Sympathy’, and ‘Haedang’, respectively (Table 1). There are 21,490 (Vital), 19,417 (Maroussia), 22,107 (Sympathy), and 24,356 (Haedang) singlets, including both un-clustered reads and reads that are clustered but are not assembled into contigs.

### Comparative analysis and functional annotation of rose flower miRNAs

The scheme used to develop functional annotation for roses is depicted in Figure 1. We downloaded genes for three sequenced Rosaceae genomes (apple, strawberry, and peach) [7] and grape [33] to compare to rose unigenes. We annotated rose unigenes (including contigs, un-assembled singlets, and un-clustered singlets) and other protein-coding genes by implementing BLASTx against non-redundant (NR)-protein and *Arabidopsis* protein sequences. We found that 51%, 61%, 58%, and 51% of unigenes aligned to known and/or hypothetical proteins for ‘Vital’, ‘Maroussia’, ‘Sympathy’, and ‘Haedang’, respectively (Table 2). Although rose unigenes were annotated at a lower level than those of related species, (i.e. 81%, 71%, 91%, and 89% of genes were annotated for apple, strawberry, peach, and grape, respectively), approximately 64-77% of rose contigs were annotated. Using Pfam, we identified 2,262, 2,315, 2,383, and 2,369 distinct domains within the ‘Vital’, ‘Maroussia’, ‘Sympathy’, and ‘Haedang’ unigenes, respectively (Table 2). These numbers are slightly less than the numbers of domains identified in apple (3,311), strawberry (3,333), peach (3,297), and grape (3,305) genes (Table 2). The reason why smaller numbers of domains were identified in *Rosa* is probably due to the fact that the genes which contain unidentified domains were expressed at lower levels in rose flowers or only expressed in non-flower tissues.

**Table 2:** Gene and unigene annotations for rose and related species

	**V**	**M**	**S**	**H**	**Apple**	**Strawberry**	**Peach**	**Grape**
**Gene/unigene**	35,657	31,434	34,725	39,722	63,541	34,809	28,702	26,346
**BLAST match**^**a**^	18,414	19,214	20,264	20,349	51,525	24,764	26,163	26,346
**Contigs**	9,062	9,425	9,395	9,816				
**Singlets**	9,352	9,699	10,869	10,533				
**Domains**^**b**^								
**Total**	10,646	11,353	11,642	11,265	72,220	36,042	37,433	32,566
**Distinct**	2,262	2,315	2,383	2,369	3,311	3,333	3,297	3,305

^a ^The number of gene or unigene annotated by BLAST search against NR database with expect value (e-value) lower than 1e^-10^.

^b ^Number of domain identified by implementing Pfam. Abbreviations: V: ‘Vital’; M: ‘Maroussia’; S: ‘Sympathy’; H: ‘Haedang’.

**Figure 2:**
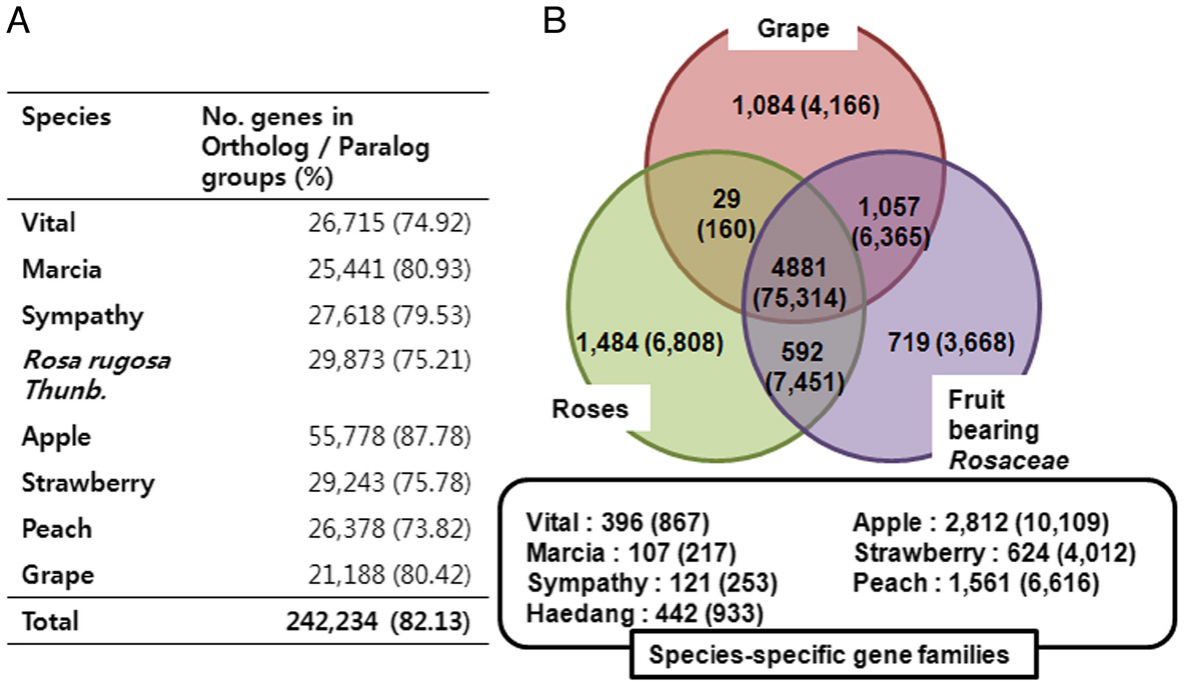
**Comparative analysis of *Rosa *gene families with genome sequenced Rosaceae (apple, strawberry, and peach) and grape.** Distribution of orthologous and paralogous gene families, including *Rosa *and apple, strawberry, peach and grape, were analyzed by OrthoMCL [34]. **A**. A total of 294,936 sequences from the eight different organisms were clustered into 242,234 (82.13%) families and singletons using OrthoMCL. **B**. Numbers of families and genes (in parenthesis) presented in each organism combination are given in the individual sections. 4,881 families (with 75,314 genes) were common to all species analyzed in this study, 592 families (with 7,451 genes) were shared in Rosaceae group. 6,808 were clustered in rose specific group with a total of 1,484 families. The species-specific gene families denote genes clustered within singe organisms.

To identify functional and evolutional relationships, we analyzed orthologs and paralogs of 294,936 gene/unigenes from 4 *Rosa*, apple, strawberry, peach and grape, by implementing orthoMCL [34]. We found that 242,234 genes were clustered into 39,447 gene families, suggesting that 82.13% of total genes or unigenes have similarity among species (Figure 2A). Approximately 23% of the 39,447 gene families were homologous to *Arabidopsis* gene families in The Arabidopsis Information Resource (TAIR) database. The TAIR provides 996 gene families and 8,331 genes, covering 32.16% of *Arabidopsis* genes [35]. Gene family comparisons provide the number of conserved and shared genes in plants available in the rose database and the number of species-specific gene families for each species (Figure 2B). On the whole, 4,881 gene families corresponding to 75,314 genes were conserved in 8 plants analyzed in this study (Figure 2B), and 592 gene families corresponding to 7,451 genes were common to all Rosaceae species, indicating that conserved functions were specific to Rosaceae (Figure 2B). In rose, 1,484 gene families with 6,808 genes were common to 4 *Rosa* and were not conserved in fruit-bearing Rosaceae species (Figure 2B). Additionally, 396, 107, 121, and 442 gene families were specific to Vital, Maroussia, Sympathy, and Haedang, respectively (Figure 2B). Of the species-specific gene families, only 3-6% was annotated based on homology to *Arabidopsis* gene families, suggesting these may be the gene families evolved within each rose.

### miRNA analysis

**Figure 3:**
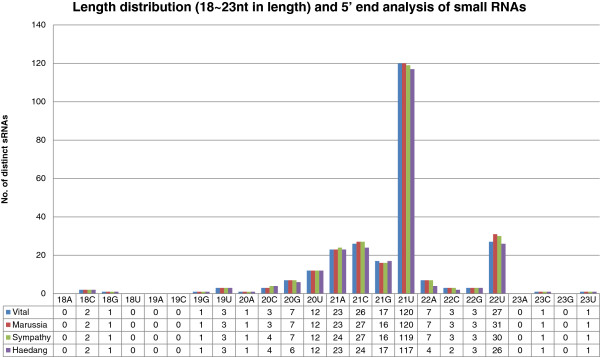
**miRNA length and first nucleotide distribution Highly abundant sRNA tags were identified/filtered according to their read length and first nucleotide. **Canonical miRNAs were 21 nt in length and began with a Uridine (U) [40].

We sequenced flower small RNA (sRNA) sequences for roses using the Illumina GAIIx platform and generated an average of 9,958,816 sRNAs per rose (Table 3). After pre-processing, 9,698,841 (94.18 %) unique sRNA sequences, with 18–26 nucleotides (nt) in length, were remained for mapping. The sRNAs were dominantly 24nt, 21nt, and 22nt in length, whose production relied on DCL3, DCL4 and DCL2, respectively (Additional file 1). [36], as previously observed in other plant species [37,38]. In order to distinguish miRNAs from all other sRNAs, we predicted secondary structures of miRNAs by mapping 9.6 million sRNA sequences to the strawberry genome because there is no genome available for rose. 3,090,334 (31.86%) sRNA tags were perfectly mapped to the strawberry genome after discarding sRNAs that are aligned to more than 10 loci in the strawberry genome. We predicted secondary structures using RNAfold, and the sequences used for this prediction were 500 bp marginal sequences from mapped sRNA. We validated miRNAs which meet the criterion of plant miRNA annotation [39]. Finally, we predicted 122, 125, 125, and 120 known and novel miRNAs for Vital, Maroussia, Sympathy, and Haedang, respectively (Table 3, Additional file 2). Among them, 21, 23, 21 and 20 miRNAs were novel miRNAs for Vital, Maroussia, Sympathy, and Haedang, respectively. (Table 3, Additional file 2). To screen additional conserved miRNAs from unmapped sRNAs, we aligned the rose sRNAs to conserved miRNAs from the miRBase [30] (Figure 1). Using similarity search, we additionally annotated 136, 137, 137, and, 128 conserved miRNAs for Vital, Maroussia, Sympathy, and Haedang, respectively, which may be missed during the mapping to the strawberry genome due to lower sequence similarities or construction of hairpin structures (Table 3, Additional file 2). Based on above analysis, we identified 84 distinct miRNAs, including 19 novel miRNAs. The size distribution of sRNA showed that approximately 98% of miRNAs fell within the range of 19–24 nt, especially abundant 21 nt in length (Figure 3). In addition, 65% and 12% of the 21 nt sRNAs had 5’ uridine (U) and adenosine (A), whose characteristic depends on Dicer cleavage and Argonaute 1 and 2 (AGO1/2) association, respectively [40,41].

**Table 3:** miRNA distribution

	**V**	**M**	**S**	**H**	
Total sRNA No.	7,440,941	11,814,817	9,210,732	11,368,773	
Trimmed sRNAs	7,339,459	11,646,177	9,049,517	11,201,170	
Unique sRNAs	2,231,008	2,520,957	2,590,637	2,955,175	
**Preprocessing**					
tRNAs	12,638	14,647	11,942	12,925	
rRNAs	50,020	70,718	49,317	56,084	
other RNAs	1,552	2,123	1,774	1,518	
Masking	2,166,798	2,433,469	2,527,604	2,884,648	
18nt to 26nt unique sRNAs after pre-processing	2,097,062	2,338,032	2,439,306	2,824,442	
18nt to 26nt total sRNAs after pre-processing	5,400,748	6,498,879	6,456,832	6,930,547	
**Total identified miRNAs**					**Total**
sRNA align to strawberry genome	754,825	919,179	815,779	600,561	
Total predicted miRNAs (Unique)	183 (122)	186 (125)	186 (125)	179 (120)	192 (130)
Conserved miRNAs in miRBase (Unique)	155 (101)	155 (102)	158 (104)	154 (100)	159 (105)
Novel miRNAs (Unique)	28 (21)	31 (23)	28 (21)	25 (20)	33(25)
Found by homology search with miRBase ^a^	136	137	137	128	137
Total miRNA groups (Major)	78 (40)	82 (42)	80 (40)	75 (41)	84 (53)
Conserved miRNA groups (Major)	63 (27)	65 (28)	65 (28)	61 (28)	65 (37)
Novel miRNA groups (Major)	15 (13)	17 (14)	15 (12)	14 (13)	19 (16)

^a ^The number of miRNAs searched by BLAST against known miRNAs in the miRBase [86]. Abbreviations: V: ‘Vital’; M: ‘Maroussia’; S: ‘Sympathy’; H: ‘Haedang’.

### Conserved miRNAs in four roses and Rosaceae

**Figure 4:**
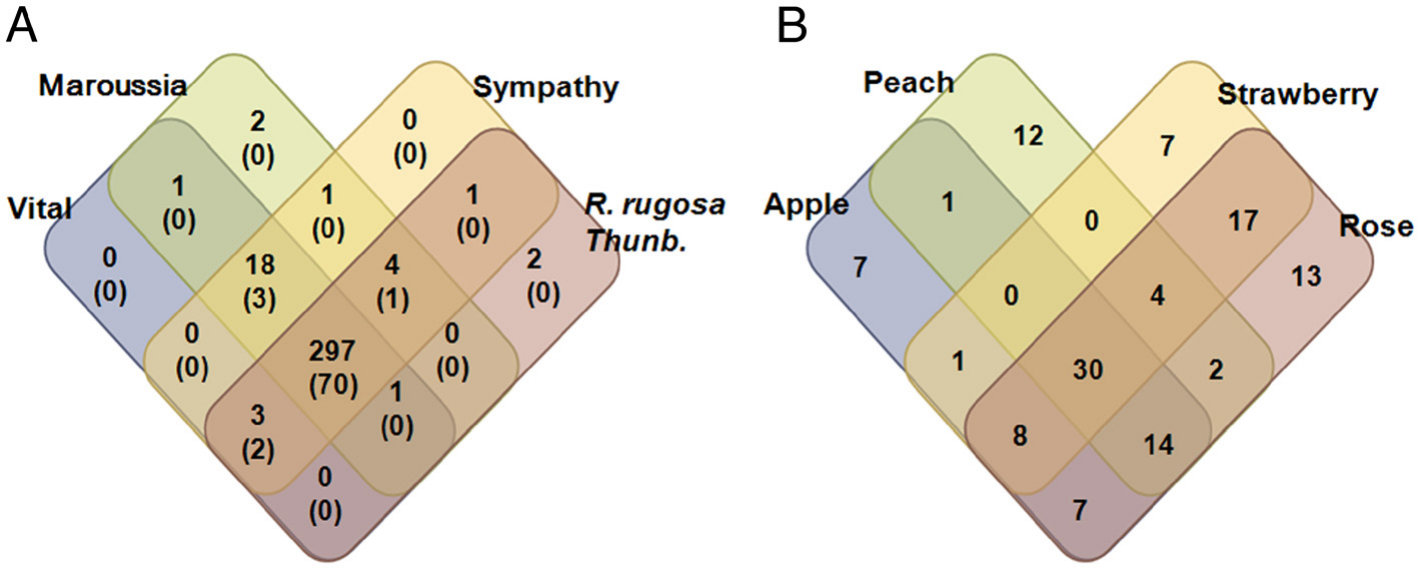
**Venn diagram showing unique and shared miRNA families. A**. Distribution of miRNA tags in the four rose cultivars (Vital, Maroussia, Sympathy, and Haedang). **B**. Distribution of miRNA tags in Rosaceae plants. The numbers of miRNA tags present in each organism combination were given in the individual sections. Venn diagram represents conservation and/or variation of miRNA tags in rose flowers by the presence or absence of miRNA tags among the four rose cultivars (A) or among Rosaceae species (B).

We investigated conservation and/or variation of miRNAs in rose flowers by analyzing the presence or absence of miRNA tags among four *Rosa* cultivars (Figure 4A). The miRNA tags denote individual miRNA with sequence redundancies. We found that two miRNA tags are uniquely expressed in Maroussia and Haedang, respectively. Most (297 miRNAs, 90%) of the miRNAs were conserved in the four Rosa cultivars (Figure 4A). We also analyzed miRNAs conserved in Rosaceae. For this analysis, we retrieved sRNA sequences from Gene Expression Omnibus (GEO) database for strawberry (GSE34813), peach (GSE18764), and apple (GSE36065). miRNAs from these species were re-analyzed according to our pipeline (Additional file 3). As a result, a total of 123 miRNAs were conserved among Rosaceae. Among them, 13, 7, 12 and 7 miRNAs were uniquely expressed in rose, strawberry, peach and apple, respectively (Figure 4B). Thirty miRNAs were conserved in all Rosaceae (Figure 4B). Strawberry has the largest number of miRNAs (17) conserved in *Rosa* flower, comparing to those in peach (2) and apple (7) (Figure 4B), which suggest that strawberry is the most closely related species of *Rosa*.

### Regulatory roles of conserved miRNAs in Rosa

**Figure 5:**
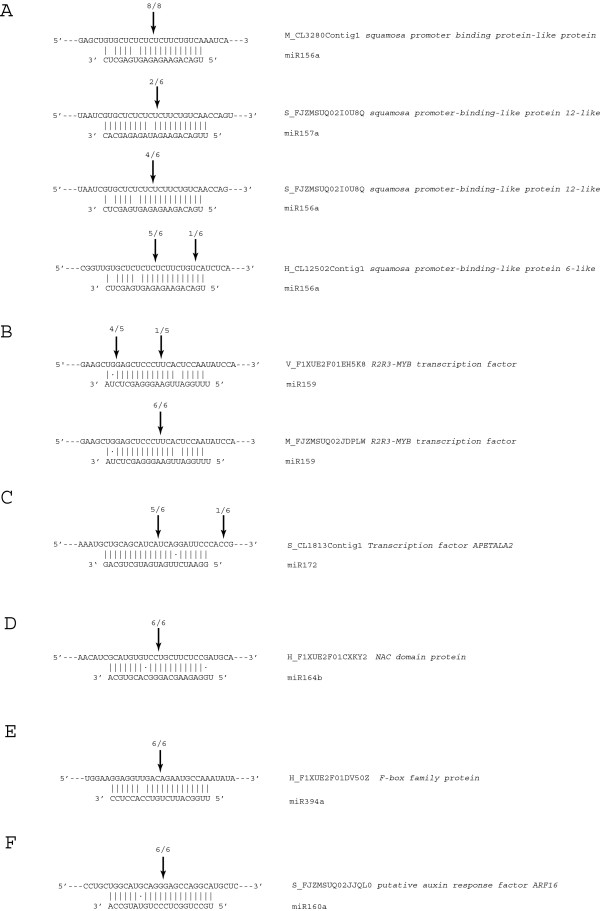
**Validation of miRNA-directed cleavage using 5’ RLM-RACE. A**-**F**. 5’ RLM-RACE PCR products terminating at a given position indicated above the each miRNA-target duplex with the frequency of clones.

Plant miRNAs are negative regulators of gene expression, and thus, play essential roles in developmental patterning [22]. Therefore, we hypothesized that the conserved miRNAs in four *Rosa* cultivars may have evolutionally conserved functions. To address this hypothesis, we analyzed conserved miRNAs whose target genes were commonly identified in *Rosa*, and miRNAs with higher reads were representatively shown (Table 4). With these criteria, we selected fifteen miRNA families, two of which were novel. These miRNAs may have conserved function, but it is necessary to experimentally validate whether these miRNAs actually regulate computationally predicted target genes. For the target validation, we selected 7 miRNA families and 15 target genes whose penalty score were four or less (Additional file 4). In the specific circumstance where the expression of a miRNA target gene is regulated through miRNA-directed cleavage mechanism, 5′ RNA ligase-mediated-rapid amplification of cDNA ends (5′ RLM-RACE) is generally employed to confirm such targeting. Therefore, it is necessary to experimentally validate whether computationally predicted targets are actually regulated by miRNAs by means of miRNA-directed cleavage of these targets because plant miRNAs regulate their target genes mainly by cleaving them [42,43]. Therefore, we isolated total RNAs from four *Rosa* cultivars and performed 5' RLM-RACE for squamosa-promoter binding protein (SBP), MYB, APETELA2 (AP2), no apical meristem (NAC), F-BOX, and auxin response factor (ARF). As a result, nine target genes of seven miRNAs were successfully validated (Figure 5). The target gene, SBP transcription factor, was validated to be target of miR156/157 families in three *Rosa* cultivars (Maroussia, Sympathy and Haedang). We observed that the SBP transcription factor was targeted both by miR156 and miR157 families in ‘Sympathy’, whereas other SBP transcription factors were targeted only by miR156 family in Haedang and ‘Maroussia’ (Figure 5A). miR156 and miR157 in plants have been grouped in one miRNA family due to their high degree of sequence similarity and their conserved target, the SBP transcription factors [22]. Additionally, for other miRNAs (miR159, miR172, miR164, miR394, and miR160 families), we confirmed miRNA-directed cleavage in one or two *Rosa* cultivars (Figure 5B-F). Considering the function of target genes, most of targets validated in 5' RLM-RACE assay were transcription factors such as SBP, MYB, AP2, ARF, and NAC transcription factors (Table 4, Figure 5), indicating that these conserved miRNAs in Rosa may have important roles in flower development.

**Table 4:** Representative targets of conserved miRNA in four roses

**miRNA**	**Sequence (5′-3′)**	**V**^**a**^	**M**^**a**^	**S**^**a**^	**H**^**a**^	**Target (UniGene)**^**b**^	PS*	**Annotation**
miR156a ^c^	UGACAGAAGAGAGUGAGCUC	1,393	1,861	2,755	3,616	H_CL12502Contig1 ^d^	2	squamosa promoter-binding-like protein
						M_CL3280Contig1 ^d^	2	squamosa promoter-binding-like protein
						S_FJZMSUQ02I0U8Q ^d^	2	squamosa promoter-binding-like protein
						V_CL1436Contig1	2	squamosa promoter-binding-like protein
miR159 ^c^	UUUGGAUUGAAGGGAGCUCUA	327,436	271,208	264,412	187,579	H_F09TDQC01DKJVB	4	U-box domain-containing protein
						M_FJZMSUQ02JDPLW ^d^	3.5	r2r3-myb transcription factor
						S_FJZMSUQ02FP8OE	4	U-box domain-containing protein
						V_F1XUE2F01EH5K8 ^d^	3.5	r2r3-myb transcription factor
miR160 ^c^	UGCCUGGCUCCCUGUAUGCCA	682	739	1,739	783	H_F1XUE2F01ECCC8	0.5	putative auxin response factor ARF16
						M_FJZMSUQ02HS2DI	0.5	putative auxin response factor ARF16
						S_FJZMSUQ02JJQL0 ^d^	0.5	putative auxin response factor ARF16
miR164b ^c^	UGGAGAAGCAGGGCACGUGCA	1,618	1,166	753	735	H_F1XUE2F01CXKY2 ^d^	3	NAC domain protein
						M_CL5581Contig1	2.5	NAC domain protein
						V_CL3217Contig1	2.5	NAC domain protein
miR167c ^c^	UGAAGCUGCCAGCAUGAUCUC	19,155	21,149	28,997	17,491	H_CL5998Contig1	4	ARF domain class transcription factor
						M_CL367Contig1	4	ARF domain class transcription factor
						S_CL788Contig1	4	ARF domain class transcription factor
						V_F1XUE2F01AFQWV	4	ARF domain class transcription factor
miR168 ^c^	UCGCUUGGUGCAGGUCGGGAA	40	145	33	98	H_F09TDQC01CTD5O	3.5	argonaute protein group
						M_CL4618Contig1	0.5	argonaute protein group
miR172 ^c^	GGAAUCUUGAUGAUGCUGCAG	2,590	1,369	1,728	720	H_CL8041Contig1	1	AP2 domain class transcription factor
						S_CL1813Contig1 ^d^	1	AP2 domain class transcription factor
						V_CL10861Contig1	1	AP2 domain class transcription factor
miR319a ^c^	UUGGACUGAAGGGAGCUCCC	392	1,117	630	518	H_F1XUE2F01EUODN	4	TCP domain class transcription factor
						M_CL839Contig1	3.5	TCP domain class transcription factor
						S_CL7921Contig1	4	TCP domain class transcription factor
						V_F1XUE2F01EH5K8	3	r2r3-myb transcription factor
miR394 ^c^	UUGGCAUUCUGUCCACCUCC	227	2,251	732	310	H_F1XUE2F01DV50Z ^d^	1	F-box family protein
						M_CL9289Contig1	1	F-box family protein
						S_CL298Contig1	3.5	conserved hypothetical protein
						V_CL3647Contig1	3.5	conserved hypothetical protein
miR396 ^c^	UUCCACAGCUUUCUUGAACUG	10,509	20,749	9,975	6,184	M_CL458Contig1	4	3-dehydroquinate dehydrogenase
						S_FJZMSUQ02FG68V	3.5	ATP binding protein
						V_CL7932Contig1	3	uncharacterized protein
miR482b ^c^	UCUUUCCUAUUCCUCCCAUCCC	2,150	5,734	4,173	6,112	H_F09TDQC01C8RL9	4	TIR-NBS-LRR resistance protein
						S_FJZMSUQ02IMY8Q	4	TIR-NBS-LRR resistance protein
						V_F1XUE2F01BNZWC	3	TIR-NBS-LRR resistance protein
miR530a	UGCAUUUGCACCUGCACCUCU	231	205	88	237	H_CL335Contig4	2.5	BZIP domain class transcription factor
						M_CL83Contig1	2.5	BZIP domain class transcription factor
						S_CL3168Contig1	2.5	BZIP domain class transcription factor
						V_CL1619Contig1	2.5	BZIP domain class transcription factor
miR894	CGUUUCACGUCAGGUUCACCA	494	325	736	239	H_CL672Contig1	3	E3 ubiquitin-protein ligase
						M_CL2017Contig1	3	E3 ubiquitin-protein ligase
						V_CL3434Contig1	3	E3 ubiquitin-protein ligase
Ng3 ^e^	UCUAAGAAACAUUCCUUGAUG	186	391	155	77	S_FJZMSUQ02GRX36	2	Probable disease resistance protein
						S_CL7967Contig1	2	Probable disease resistance protein
						V_CL9334Contig1	4	Probable disease resistance protein
Ng11 ^e^	GUGGAGUUCUGGGAAAGAAG	51	8	2	7	H_CL10098Contig1	3.5	Matrix metalloprotease 1
						M_CL1633Contig2	4	Acyltransferase-like protein
						S_CL7901Contig1	3.5	Metalloendoproteinase 1-like

^a^ numbers denote normalized expression value in each *Rosa*. ^b^ A single capital letter in front of the UniGene ID represent the abbreviations of Rosa. For example, “M_CL3280Contig1” is unigene in ‘Maroussia’. All abbreviations of tag (V, M, S, H) are ‘Vital’, ‘Maroussia’, ‘Sympathy’, and ‘Haedang’, respectively. ^c^ miRNAs identified in strawberry libraries which downloaded from GEO (Accession No. GSE34813). ^d^ target genes successfully validated using 5’RACE (Figure 5). ^e^ Novel miRNAs identified in this study.

### miRNAs putatively determine the colour of rose

Flavonoids synthesize diverse secondary metabolites determining flower colour such as antocyanins, flavonols, flavones and proantocyanidins. We aligned *Rosa* unigenes against *Arabidopsis* genes involved in flavonoid or carotenoid biosynthetic pathways. Subsequently, we analyzed miRNAs whose target genes are related to those pathways. We identified thirteen miRNA families putatively targeting 27 target genes (Table 5). To analyze miRNAs regulating colour-related pathways, we examined miRNA enrichment in given *Rosa* with p-values less than 0.001 by applying Audic’s test (Table 5). The miRNA enrichment means that statistically over-expressed miRNA tags in a given rose cultivar comparing with miRNA tag constructed in another cultivar. Five of the miRNAs (miR171, miR166i, miR159e, miR845, and miR396e) were enriched in the white flowers of Maroussia. Especially, miR396e, which is predicted to target Cytochrome P450 (CYP), had two fold higher read counts than other roses. CYP is an essential gene for synthesizing red colour in flowers, fruit, and epidermal tissues [44,45]. It is suggested that these miRNAs may negatively regulate target genes to prevent accumulation of carotenoids or flavonoids, resulting in white flowers. However, no target genes with penalty scores of four or less were predicted. Thus, we were not able to confirm the miRNA-directed cleavage of target genes involved in colour determination. However, among the target genes, the SPL and R2R3-MYB transcription factors, both of which are known to negatively regulate flavonoid biosynthesis, were experimentally validated to be targets of miR156 and miR159, respectively [46,47]. In addition, the target gene of miR159 was predicted only in Maroussia (white) and Haedang (pink), which indicates that the colours of the rose flowers, may be tightly regulated via complex mechanism of various miRNAs in nature (Additional file 5).

**Table 5:** miRNAs putatively targeting flavonoid biosynthetic pathway

**miRNA**	**Sequence (5'-3')**	**V**	**M**	**S**	**H**	**Target (UniGene)**	**Score**	**Annotation**
miR171	UUUUUCUGAUUGAGCCGUGCC	17	**51**	19	6	S_CL1522Contig1	5.5	Anthocyanin synthase (ANS)
miR858b	UUCGUUGUCUGUUCGACCUGA	39	39	19	**225**	M_CL545Contig1	4	HTH_MYB
miR535	UGACGAUGAGAGAGAGCACGC	**67,950**	30,364	24,750	**51,647**	S_CL160Contig1	6	Flavone synthase (FNS)
miR166i	UGAAUGUCGUCUGGUUCGAAA	**106**	**80**	68	51	S_CL5764Contig1	6	Flavonol synthase (FLS)
miR477b	ACUCUCCCUCAAGAGCUUCUAG	12	50	**154**	**213**	H_CL379Contig1	6	Dihydroflavonol-4-reductase (DFR)
miR159e	UUUGGCUUGAAGGGAGCUCUA	78	**80**	53	51	V_F1XUE2F01CHQVJ	6	Cytochrome P450
						S_CL2652Contig1	6	Cytochrome P450
miR172c	GUAGCAUCAUCAAGAUUCAC	7	4	2	**58**	H_CL3307Contig1	6	Cytochrome P450
						S_CL373Contig2	6	Cinnamate 4-hydroxylase (C4H)
						V_CL1167Contig1	6	Cinnamate 4-hydroxylase (C4H)
miR399	GGGCGUCUUUCCUUUGGCAGG	16	0	1	**42**	S_CL3909Contig1	6	Cytochrome P450
miR396a	CACAGCUUUCUUGAACUU	11	22	**43**	7	H_CL735Contig1	5.5	Chalcone and stilbene synthase
						H_F09TDQC01AFYKY	5.5	Chalcone synthase (CHS)
						M_FJZMSUQ02GLZSQ	6	Short chain alcohol dehydrogenase
						S_CL831Contig1	5.5	Chalcone and stilbene synthase
						V_CL2603Contig1	5.5	Constitutive photomorphogenic DWARF (CPD)
						V_CL3087Contig1	5	Cytochrome P450
						V_F1XUE2F01B3HEY	5	Cinnamate 4-hydroxylase (C4H)
miR845e	ACCUGGCUCUGAUACCAAUUG	84	**387**	63	**947**	H_CL735Contig1	5.5	Chalcone and stilbene synthase family protein
						S_CL831Contig1	5.5	Chalcone and stilbene synthase family protein
miR396e	UUCCACAGCUUUCUUGAACUU	6,309	**13,690**	**7,655**	2,520	H_CL11974Contig1	6	Cytochrome P450
miR319	UGGACUGAAGGGAGCUCCC	7	9	2	**151**	M_FJZMSUQ02HT34C	4.5	Cytochrome P450
miR396b	CACAGCUUUCUUGAACUG	20	39	**62**	22	S_FJZMSUQ02HBX1V	6	Cytochrome P450
						V_CL10716Contig1	6	CPD (CONSTITUTIVE PHOTOMORPHOGENIC DWARF)
						V_CL1787Contig1	6	Flavone synthase (FNS)
						V_CL2603Contig1	6	CPD (CONSTITUTIVE PHOTOMORPHOGENIC DWARF)
						V_F1XUE2F01B3HEY	6	C4H (CINNAMATE-4-HYDROXYLASE)

The bold characters denote enriched miRNA tags with less than p<0.001 by analyzing Audic’s test [88]. Abbreviations: V: ‘Vital’; M: ‘Marcia’; S: ‘Sympathy’; H: ‘Haedang’.

In addition to miRNA profiles, we also analyzed differential expression of colour-related genes involved in carotenoid and flavonoid biosynthesis (Table 6). We identified 28 (with 68 reads), 15 (15), 31 (76) and 28 (100) unigenes for carotenoid biosynthesis ( Additional file 6) and 31 (96), 12 (12), 40 (232), and 38 (201) Unigenes for flavonoid biosynthesis (Additional file 7) for Vital, Maroussia, Sympathy and Haedang, respectively. Based on these data, the number of colour-related unigenes in Maroussia is lower than other roses and fruit-bearing Rosaceae. Moreover, all colour-related unigenes in Maroussia were singlets due to smaller number of sequence reads, whereas unigenes in other roses were assembled into contigs. In summary, we found that some of the miRNAs were enriched in Maroussia, and the smaller number of sequencing reads of colour-related genes were found in Maroussia, which raises an intriguing possibility of miRNA-directed regulation of colour-related gene in Maroussia.

**Table 6:** Transcript profiles for colour-related genes

	**V**	**M**	**S**	**H**	**Apple**	**Strawberry**	**Peach**	**Grape**
**Carotenoid biosynthesis**								
**Gene/unigene**	28	15	31	28	66	35	25	30
**Number of reads**	68	15	76	100				
**Average reads per unigene**	2.4	1	2.4	3.6				
**Flavonoid biosynthesis**								
**Gene/unigene**	31	12	40	38	159	55	34	63
**Number of reads**	96	12	232	201				
**Average reads per unigene**	3.1	1	5.8	5.2				

Abbreviations: V: ‘Vital’; M: ‘Maroussia’; S: ‘Sympathy’; H: ‘Haedang’.

## Discussion

Rose breeders select roses according to particular criteria, which include cold and disease resistance, flower form, recurrent flowering, and to some degree, scent [21]. In spite of the importance of phenotypes for roses, only a few studies have addressed flower features at the molecular level. Prior to this study, only a few thousand rose unigenes had been deposited in the EST database (dbEST) at NCBI (search query: "*Rosa*"[Organism]). With the aim of providing valuable resources for molecular studies of rose flowers, we constructed the Rose transcriptome database and analyzed miRNA sequences from three *R. hybrids* (Vital, Maroussia, and Sympathy) and Haedang.

These rose cultivars were selected because of a range of structures (from a single to multiple layers of petals) and colours (from white to dark red) in their flowers (Figure 1). The database includes 35,657, 31,434, 34,725, and 39,722 unigenes for ‘Vital’, ‘Maroussia’, ‘Sympathy’, and ‘Haedang’, respectively (Table 1), covering an average of 55.96%, 60.70%, 59.14%, and 61.57% of apple, strawberry, peach, and grape protein-coding genes, respectively. We annotated unigenes up to similar levels of related-species, especially in contigs (Table 2). However, overall numbers of identified domains were slightly lower than those identified in related species (Table 2). These domains may be expressed at lower levels in rose flowers or not expressed in flower tissues. In addition, the Rose database provides additional information for the unigenes, including gene description, domains, GO, PO, metabolic pathway, FunCat, and homologous gene families among roses, Rosaceae families (apple, strawberry, and peach) and grape (Figure 1). The sequence dataset and high-quality annotation enabled us to analyse and compare the transcripts which are specialized in rose flowers and related species.

With the help of large-scale sRNA sequencing, a large number of miRNAs have been identified, characterized, and reported [48-54]. However, these techniques are best applied to plant species whose genomes have been assembled, annotated and published (i.e., *Arabidopsis*, Rice and Grape [51,52,54]) because it is necessary to predict and construct hairpin precursors of potential miRNAs, using neighbouring genomic sequences of the mapped sRNAs to distinguish high quality miRNAs from other sRNAs [24,28]. Therefore, in the absence of the genome sequences, the strategy to identify miRNAs is limited [55,56].

In this study, we set out to identify highly qualified conserved and novel miRNAs from floriculture plants *Rosa* × *hybrida* (Vital, Maroussia, and Sympathy) and Haedang, for which genomes are not available. To predict hairpin precursors for potential miRNAs, we mapped sRNA tags to the strawberry genome, the most closely related species among genome sequenced plants, and identified mature miRNAs using modified miRDeep, a program that employs a probabilistic model of miRNA biogenesis [24,29]. Mapping sRNA to cross-species is possible because secondary structures for pre-miRNAs are highly conserved between species [22]. Identification of miRNAs by mapping of sRNA to related species has strengths in that it i) maximizes the screening of novel miRNAs in plants for which genome is not available, ii) allows identification of the conserved and novel miRNAs between related species, and iii) provides an incremental improvement in accuracy of miRNA identification by utilizing secondary structures. We might have missed some novel miRNAs that are specific to roses; however, these novel miRNAs are less likely to be important in functional studies because they are expressed at low levels and lack target-genes, whereas conserved miRNAs are usually highly expressed [23,57].

With this approach, we identified 122, 125, 125, and 120 miRNAs for ‘Vital’, ‘Maroussia’, ‘Sympathy’, and ‘Haedang’, respectively (Table 3). Of these miRNAs, 21, 23, 21, and 20 were novel miRNAs for ‘Vital’, ‘Maroussia’, ‘Sympathy’, and ‘Haedang’, respectively (Table 3). These novel miRNAs correspond to 16–18% of the total identified miRNAs. We also identified 101, 102, 104, and 100 conserved miRNAs by aligning sRNA tags to known miRNAs for ‘Vital’, ‘Maroussia’, ‘Sympathy’, and ‘Haednag’, respectively (Table 3). This hybrid method maximized the identification of expressed miRNAs for rose flowers, therefore providing many miRNA candidates (Figure 1). In addition, this allows us to understand the conserved and unique regulatory processes occurring in rose flowers of different species and hybrids.

miRNA research is advancing from analysis of single tissue or species to comparison of miRNAs between species [50], varieties [58], tissues [48,51-53,59], or different development stages [54]. Today, NGS technologies allow for the comparison of miRNA profiles with statistical analysis of the redundancy of miRNA tags and enables discussion of tissue- or organ-specific miRNAs [51,52,54,58,59]. We first compared miRNA profiles among three *R. hybrida* cultivars and Haednag to analyze the conservation and variation of miRNA tags in the four roses (Figure 4). We identified miRNA tags detected in single rose cultivar, leading to specific function to *Rosa*. We also identified 297 conserved miRNAs in all four roses (Figure 4A), including 274 known miRNAs and 23 novel miRNAs. Among 274 conserved miRNA, 70 miRNA tags were also identified in strawberry. In addition, 30 conserved miRNAs were also conserved in all Rosaceae (Figure 4B), suggesting that Rosaceae share many conserved miRNAs between ornamental and fruit-bearing plants. Furthermore, among the conserved miRNAs, seven miRNA families were experimentally confirmed to regulate their target genes by cleavage mechanism using 5' RACE assay (Figure 5), suggesting that the conserved miRNAs identified in this study are actually functional.

Most of the target genes validated in this study were transcription factors (Figure 5A-D, F), and their mutant phenotypes were characterized in many model plants (Table 4). Over-expression of miR156 (Figure 5A) and miR159 (Figure 5B) induced delayed flowering in *Arabidopsis* by negatively regulating SPL and MYB family transcription factors genes, respectively [60,61]. The expression of miR167-resistant ARF6 (Figure 5A) leads to arrested ovule development and indehiscent anthers [62]. miR172 (Figure 5C) is crucial for development of reproductive organs and for timely termination of floral stem cells by regulating AP2 RNA stability [63]. The expression of miR172-resistant AP2 induces the formation of variable numbers of floral organs with numerous petals and lacking inner whorl organs [63,64]. miR160 regulates development by altering expression of auxin-induced genes through ARF families [65,66]. miR164 targets CUC1 and CUC2 transcripts in *Arabidopsis* and controls leaf margin development. Therefore, we assumed that the conserved miRNAs, which were experimentally validated in this study, may have important roles in floral organ identity or flower developments.

In addition, these eight miRNAs are evolutionary conserved and abundantly expressed miRNAs in roses. According to previous studies, miR156, miR159, and miR160 are evolutionary conserved in all land plants, and miR164, and miR172 are conserved in seed-bearing plants [57]. Evolutionarily conserved miRNAs in plants tend to regulate ancestral transcription factors that specify basic meristem functions, organ polarity and separation, cell division, or hormonal control (reviewed by Garcia [67]). Based on experimental validation of conserved miRNAs and the current discussions [22,23,67], we might expect that novel and un-validated miRNAs identified in this study (Table 4) possibly play important roles such as flower development or hormonal control. Our analysis suggests that the Rose database is a useful tool to search for candidate target transcripts or miRNAs that play roles in flower development in rose and for those have a variety of other specific functions.

Flower colour in most angiosperms is one of the most important targets for plant breeders and many different-coloured cultivars have been bred using natural mutants or genetically-related species. Flower colours are determined by an accumulation of secondary metabolites such as flavonoids, carotenoids, and betalains [68,69]. We examined miRNA profiles in which target genes were involved in colour-metabolite related biosynthesis pathways to gain insight into the regulation of the white flowered cultivar, Maroussia (Figure 1), which seems to be regulated by miRNAs (Table 5). We hypothesized that miRNA enrichment in Maroussia may negatively regulate the colour-related genes, leading to white colour of Maroussia. Although miRNA enrichments were also observed in other *Rosa* (Vital, Sympathy, Haedang), the most interesting thing was that five miRNA tags were enriched in Maroussia. Especially, miR356e was expressed up to five times more than other Rosa. Thus, the function of its target, CYP, which involves catalyzing the biosynthesis of flavonoids and cyanidin (red to magenta) and delphinidin (violet to blue) [44,45], would be more negatively regulated in Maroussia, which may possibly lead to lack of colour . Unfortunately, we were not able to validate miRNA-directed cleavage of these targets due to high penalty score of the target genes (Table 5). However, we would rather expect that miRNA-directed regulation of target gene possibly lead to low read counts of target genes (i.e. low level of expression) in the transcriptome library, which makes target identification more challenging.

However, previous studies reported that two transcription factors, SPL and R2R3-MYB, both of which regulate expression of antocyanins-related genes. Moreover, over-expressed miR156 directly prevent the expression of anthocyanin biosynthetic genes (Additional file 5) by targeting SPL9, in *Arabidopsis*[47]. In this study, we identified nine miR156 members from all *Rosa* (Additional file 2), and their target genes, SPL transcription factors, were experimentally validated by 5’ RACE assay (Figure 5). The miR159 were among the most frequent in our library (187,579; 271,208; 264,412 and 327,436 for ‘*R.thunb.*’, ‘Marcia’, ‘Sympathy’, and ‘Vital’, respectively) and its sequencing frequencies were 10 to 100 times more than other relatively abundant miRNA families, including miR156, miR157, and miR167 (Table 4). Along with this, it has been previously reported that differential expression of *R2R3-MYB* gene determine colour patterning in plants that are linked with anthocyanin production [70,71]. Given the fact that the miR159 is very highly expressed in *Rosa* and miR159-directed cleavage of *R2R3-MYB* gene is confirmed using 5' RACE, our results raise an intriguing possibility that miRNAs in roses may be involved in pigment synthesis pathway. In addition, miR828, and miR858, which are also involved in R2R3-MYB regulation[72] was predicted target MYB genes in Rosa (Additional file 2).

Based on the miRNA profiles (Table 5, Additional file 5) and the previous research, flower colour seems to be regulated by combinatorial mechanisms. Therefore, it is difficult to elucidate the molecular mechanism or miRNA-directed regulation of colour determinacy of *Rosa* flowers. Nevertheless, miRNA profiles give potential clues to examine the colour of *Rosa* flowers. Moreover, there are limited numbers of unigenes currently available. Therefore, we would expect to reveal more direct evidence of miRNA-mediated regulation of colour development in *Rosa* when genome resources become more available.

In addition to the miRNA profiles, we also compared the expression profiles of colour-related transcripts in *Rosa* and Rosaceae families (Table 6). It shows that Maroussia contains less number of unigenes (similar to strawberry or peach) than other *Rosa* cultivars. The higher number of colour-related gene in apple arose by current whole genome duplication [2]. In addition, the average number of reads per gene in Maroussia was also smaller than other *Rosa* cultivars because all of them were singlets (Table 6). On the whole, based on expression profiles of miRNA and the smaller number of transcripts involved in colour-metabolite related biosynthesis in Maroussia, it is possible to expect that colour-related genes are systemically repressed in Maroussia.

## Conclusions

The Rose database is the first database that allows for the comparison of transcripts and miRNAs for the flowers of *Rosa hybrida* and Haedang, containing more than 30,000 transcript sequences for each rose and more than 300 conserved and novel miRNAs with high quality annotations. These data and analysis are available at http://210.218.199.249/rose/. We identified several miRNA families that are conserved and highly expressed in four *Rosa*, including novel miRNAs. We also present preliminary data which raises intriguing possibilities of miRNA-directed regulation in the white colouration of the Maroussia flower. Our study demonstrates that the Rose database may facilitate a comprehensive understanding of rose flower development, and is a valuable genetic resource for the analysis of gene functions and regulatory pathways that determine flower phenotypes.

## Methods

### Rose flower materials and RNA preparation

The flowers of two red roses (Vital and Sympathy) and white one (Maroussia) at various developmental stages (from bud to open flower) were purchased from a rose farm located in Goyang, GyeongGi-Do province, South Korea. Flowers at various developmental stages from vegetatively propagated *R. rugosa* Thunb were collected from natural habitats.

A modified Suzuki’s method [73] was used for total RNA extraction as follows: Rose flowers were ground to a fine powder in liquid nitrogen using a mortar and pestle. The powder was transferred to a tube containing extraction buffer (100 mM Tris–HCl pH 6.8, 25 mM EDTA pH 8.0, 2% (w/v) cetyltrimethylammonium bromide, 1.4 M NaCl, 2% (w/v) polyvinyl polypyrrolidone, 7% (v/v) 2-mercaptoethanol, and 0.5 g/l spermidine trihydrochloride) pre-heated to 65°C in a powder-to-buffer ratio of 1:3 to 1:5. The sample was mixed rapidly and incubated for 5 min at 65°C then cooled to room temperature. An equal volume of chloroform was added to the homogenate followed by vortexing for 1 min. The mixture was centrifuged at 12,000 rpm for 10 min at 4°C and the upper phase was transferred to a new tube. Chloroform extraction was repeated 4–6 times until the middle layer was clear. Then, 0.6 volumes of 10 M LiCl was added to the sample before incubation at −20°C for 1 h and subsequent centrifugation at 10,000 rpm for 20 min at 4°C. Pellets were resuspended with DEPC-treated water and 1/10 volume of 3 M sodium acetate (pH 5.5) and an equal volume of isopropanol was added before centrifugation at 12,000 rpm for 30 min at 4°C. The precipitated total RNA pellet was washed with 75% ethyl alcohol, air-dried for 10 min, and dissolved in DEPC-treated water.

### Sequencing of cDNA and small RNA

A total of 2 μg of mRNA isolated from total RNA using a PolyATract® isolation system (Promega, Medison, WI, USA) was converted to cDNA using the cDNA Synthesis Kit (Stratagene, La Jolla, CA, USA). Approximately 5 μg of cDNA was sheared by neubulization to produce random 300–800 bp fragments. Oligonucleotide adaptors were ligated to the fragmented cDNA samples. Fragments were denatured to generate single-stranded DNA that was amplified by emulsion PCR for sequencing. Sequencing was performed on a 454 GS FLX system (Roche, Fresno, CA, USA) at NICEM (Seoul National University, Korea; http://nature.snu.ac.kr). The raw data submitted to NCBI under accession no. SRA049095.

Small RNA libraries were constructed as described previously [74] with some modifications. Low molecular weight RNA was isolated from 200 mg of total RNA by PEG 8000/NaCl precipitation. Small RNAs (20–30 nt) were purified from 15% denaturing PAGE gels and ligated first with the 5’ RNA adaptor and then with the 3’ RNA adaptor provided by Illumina. In each step, the ligated product was PAGE-gel purified. After first-strand synthesis and 18 cycles of PCR amplification, the product was PAGE-gel purified and submitted for sequencing on an Illumina GAIIx at MACROGEN (Seoul, Korea; http://www.macrogen.com). The raw data and miRNA profiles submitted to NCBI under accession no.GSE39882.

### Pre-processing and assembly of rose transcripts

In a pre-processing step, we masked low-complexity and poly (A/T) sequences, and removed reads less than 100 bp using the SeqClean program (downloaded from the Dana Farber Cancer Institute; http://compbio.dfci.harvard.edu/tgi/software/). For contig assembly, we clustered reads into groups using megablast with criteria of more than 30 bp alignments between reads and identities greater than 94%. We then applied CAP3 to assemble the clustered reads into contigs [32].

### Transcript annotation and comparative analysis

The overall process and integrated databases are represented in Figure 1. To analyze functional annotation for each transcript, we aligned contig and singlet sequences with a lower expect value of 1e^-10^ against plant non-redundant (NR) proteins downloaded from the NCBI database [75] and against *Arabidopsis* proteins downloaded from TAIR (ver. 10; http://www.arabidopsis.org/) [76]. Based on the *Arabidopsis* annotation, we integrated pathway information referring to KEGG [77], FunCat [78], and plant ontology (PO) [79,80]. Domains were identified with hmmPfam in the InterProScan Package [81]. Gene ontology (GO) was also analyzed based on the hmmPfam annotation. To exploit functionally related genes among related species, we downloaded protein and CDS sequences of the Rosaceae family (apple, strawberry, and peach), from GDR (http://www.rosaceae.org/, [7]), and grape, from Grape Genome Browser (http://www.genoscope.cns.fr/externe/GenomeBrowser/Vitis/, [33]), and analyzed orthologs and paralogs with OrthoMCL [34].

### miRNA analysis and miRNA target prediction

The scheme used for identifying miRNAs is presented in Figure 1. We consulted Breakfield et. al. paper for miRNA prediction [37]. To get high-quality small-RNA (sRNA) reads, we removed poor quality reads and adaptor sequences (5’-ATCTCGTATGCCGTCTTCTGCTTG-3’) using the FASTX toolkit (downloaded from http://hannonlab.cshl.edu/fastx_toolkit/download.html, [82]), from raw data (Table 3). We removed sRNA sequences shorter than 18 nt in length or containing ambiguous nucleotides. From cleaned sequences, we further removed sRNAs aligned to the non-coding RNAs (ncRNA) such as rRNAs, tRNAs, snRNAs and snoRNAs. We collected ncRNAs from Rfam [83], the Plant snoRNA Database [84], and 19,350 rRNAs from the NCBI database. tRNAs were predicted with tRNAScan-SE [85] for the strawberry reference genome. We predicted tRNAs from strawberry because it was used to predict secondary structure of miRNA. The remaining sRNAs were aligned to strawberry genomes. We used sRNAs for further analysis with following criterion 1) perfect match, 2) less than 10 locations of the strawberry genome. The secondary structures were constructed with RNAfold (for sRNAs more than 25 copy numbers). From validated sRNA sequence, we predicted proper mature-star hairpin pairing with 500 bp marginal sequence from mapped sRNAs. Among the sRNAs with the same 5’ alignments, we selected representative sRNA with maximum read. We selected the mature-star sequences within the 15 bp margin and refolded by RNAfold. We applied a modified miRDeep program to predict *Rosa* miRNAs [24] and selected valid miRNAs suitable for plant miRNA annotation [39]. We confirmed conserved miRNA by searching homologous miRNAs in miRBase (http://www.mirbase.org/, ver. 17, [86]) and grouped miRNA families. During the sRNA mapping to the strawberry genome, several putative miRNAs were removed because of lower similarity. Adding these miRNAs, we identified conserved miRNAs by aligning unmapped sRNAs to the miRNA sequences in miRBase. Target genes for miRNAs were predicted using the web-based TargetFinder program (http://carringtonlab.org/resources/targetfinder, [87]).

### miRNA target validation assays

For miRNA target validation, gene-specific 5′ RNA ligase-mediated rapid amplification of cDNA ends (5′ RLM-RACE) was performed using the GeneRacer Kit (Invitrogen). 1.5μg of total RNAs from *R. thunb*, Marcia, Sympathy and Vital were ligated to 0.25μg of the GeneRacer RNA oligo adapter (5’–CGACUGGAGCACGAGGACAC UGACAUGGACUGAAGGAGUAGAAA-3’). The combination of oligo(dT) and random hexamers were then used to prime 1^st^ strand cDNA synthesis in a reverse transcription reaction. The resulting cDNA was PCR-amplified with GeneRacer 5’ primer (5’-CGACTGGAGCACGAGGACACTGA-3’) and each respective gene-specific primer (shown in Additional file 4). The PCR product was further amplified by nested PCR using GeneRacer 5’ nested primer (5′-GGACACTGACATGGACTGAAGGAGTA-3′) and each respective gene-specific primer (shown in Additional file 4). The final PCR product was gel-purified and finally cloned into TA vector (RBC Bioscience) for sequencing.

### miRNA profile analysis

Audic’s test is statistic analysis which often applied to analyze digital gene expression profiles [88]. We applied this test to estimate miRNA profiles for each *Rosa*. Read count of each identified miRNA is normalized to the total number of miRNA read counts that are matched to the reference genome or known miRNA in each sample. The expression of miRNAs were represented by the number of reads (or redundancies) of the same miRNA tag (or sequence). We hypothesized that miRNA is likely to be specifically abundant in *Rosa*, when a miRNA tag has a large number of reads derived from a given *Rosa* cultivar as compared to another *Rosa* cultivar. We applied Audic’s test to estimate the probability of differential expression for individual miRNA between two pools (a specific *Rosa* cultivar versus other *Rosa* cultivars) of reads [88]. Details were denoted in Additional file 8 with the 2 * 2 contingency table and the probability equation for Audic’s test [88].

## Competing interests

The authors declare that they have no competing interests.

## Authors' contributions

JK designed the algorithm, carried out the majority of the analyses. JHP designed and performed majority of 5' RACE experiments. JYL performed the computational analysis of microRNAs. BL and JC carried out all web programming. CL, JR, WK, and HL prepared the RNA and cDNA from roses. YC and DK performed the experiments. JK, JHP, CS, and SK wrote the manuscript. CH, YN, CS, and SK supervised the project and participated in the design of the database. All authors read and approved the final manuscript.

## Supplementary Material

Additional file 1 The statistics of sRNA and its distribution. Click here for file

Additional file 2 **Raw data of miRNAs identified in Vital, Maroussia, Sympathy, and Haedang. **This file provides mapped known/novel and unmapped known miRNAs. In addition, major miRNAs with miRNA/miRNA* pairs were also provided. Click here for file

Additional file 3 **The miRNA lists conserved in Rosaceae families. **miRNA libraries for strawberry, peach, apples were downloaded from GEO database and re-analyzed. Click here for file

Additional file 4 Gene-specific primers used for target validation using 5' RACE. Click here for file

Additional file 5 **Schematic diagram of flavonoid biosynthetic pathways and miRNA profiles targeting flavonoid biosynthetic genes. **Colours represent enrichment genes in specific *Rosa *measured by applying Audic’s test (p value < 0.001).* miRNA cleavage were experimentally validated (Figure 5). Click here for file

Additional file 6 Gene list involved in Carotenoid pathway. Click here for file

Additional file 7 Gene list involved in Flavonoid pathway. Click here for file

Additional file 8 The 2 * 2 contingency table and the probability equation for Audic’s test. Click here for file

## References

[B1] Shulaev V Korban SS Sosinski B Abbott AG Aldwinckle HS Folta KM Iezzoni A Main D Arus P Dandekar AM Multiple models for Rosaceae genomics Plant Physiol 2008 147 3 985 1003 10.1104/pp.107.115618 18487361 PMC2442536

[B2] Velasco R Zharkikh A Affourtit J Dhingra A Cestaro A Kalyanaraman A Fontana P Bhatnagar SK Troggio M Pruss D The genome of the domesticated apple (Malus x domestica Borkh) Nat Genet 2010 42 10 833 839 10.1038/ng.654 20802477

[B3] Shulaev V Sargent DJ Crowhurst RN Mockler TC Folkerts O Delcher AL Jaiswal P Mockaitis K Liston A Mane SP The genome of woodland strawberry (Fragaria vesca) Nat Genet 2011 43 2 109 116 10.1038/ng.740 21186353 PMC3326587

[B4] Debener T Mattiesch L Construction of a genetic linkage map for roses using RAPD and AFLP markers Theoret Appl Genet 1999 99 5 891 899 10.1007/s001220051310

[B5] Spiller M Linde M Hibrand-Saint Oyant L Tsai CJ Byrne DH Smulders MJ Foucher F Debener T Towards a unified genetic map for diploid roses Theor Appl Genet 2011 122 3 489 500 10.1007/s00122-010-1463-x 20936462

[B6] Gar O Sargent DJ Tsai CJ Pleban T Shalev G Byrne DH Zamir D An autotetraploid linkage map of rose (Rosa hybrida) validated using the strawberry (Fragaria vesca) genome sequence PLoS One 2011 6 5 e20463 10.1371/journal.pone.0020463 21647382 PMC3103584

[B7] Jung S Staton M Lee T Blenda A Svancara R Abbott A Main D GDR (Genome Database for Rosaceae): integrated web-database for Rosaceae genomics and genetics data Nucleic Acids Res 2008 36 Database issue D1034 D1040 17932055 10.1093/nar/gkm803 PMC2238863

[B8] Foucher F Chevalier M Corre C Soufflet-Freslon V Legeai F Hibrand-Saint Oyant L New resources for studying the rose flowering process Genome/National Research Council Canada = Genome/Conseil national de recherches Canada 2008 51 10 827 837 18923534 10.1139/G08-067

[B9] Guterman I Shalit M Menda N Piestun D Dafny-Yelin M Shalev G Bar E Davydov O Ovadis M Emanuel M Rose scent: genomics approach to discovering novel floral fragrance-related genes Plant Cell 2002 14 10 2325 2338 10.1105/tpc.005207 12368489 PMC151220

[B10] Dubois A Remay A Raymond O Balzergue S Chauvet A Maene M Pecrix Y Yang SH Jeauffre J Thouroude T Genomic approach to study floral development genes in Rosa sp PLoS One 2011 6 12 e28455 10.1371/journal.pone.0028455 22194838 PMC3237435

[B11] Mahomed W Berg N EST sequencing and gene expression profiling of defence-related genes from Persea americana infected with Phytophthora cinnamomi BMC plant biology 2011 11 167 10.1186/1471-2229-11-167 22108245 PMC3233532

[B12] Blair MW Fernandez AC Ishitani M Moreta D Seki M Ayling S Shinozaki K Construction and EST sequencing of full-length, drought stress cDNA libraries for common beans (Phaseolus vulgaris L) BMC plant biology 2011 11 171 10.1186/1471-2229-11-171 22118559 PMC3240127

[B13] Tillett RL Ergul A Albion RL Schlauch KA Cramer GR Cushman JC Identification of tissue-specific, abiotic stress-responsive gene expression patterns in wine grape (Vitis vinifera L.) based on curation and mining of large-scale EST data sets BMC plant biology 2011 11 86 10.1186/1471-2229-11-86 21592389 PMC3224124

[B14] Deveshwar P Bovill WD Sharma R Able JA Kapoor S Analysis of anther transcriptomes to identify genes contributing to meiosis and male gametophyte development in rice BMC plant biology 2011 11 78 10.1186/1471-2229-11-78 21554676 PMC3112077

[B15] Venglat P Xiang D Qiu S Stone SL Tibiche C Cram D Alting-Mees M Nowak J Cloutier S Deyholos M Gene expression analysis of flax seed development BMC plant biology 2011 11 74 10.1186/1471-2229-11-74 21529361 PMC3107784

[B16] Mondego JM Vidal RO Carazzolle MF Tokuda EK Parizzi LP Costa GG Pereira LF Andrade AC Colombo CA Vieira LG An EST-based analysis identifies new genes and reveals distinctive gene expression features of Coffea arabica and Coffea canephora BMC plant biology 2011 11 30 10.1186/1471-2229-11-30 21303543 PMC3045888

[B17] Mardis ER Next-generation DNA sequencing methods Annu Rev Genomics Hum Genet 2008 9 387 402 10.1146/annurev.genom.9.081307.164359 18576944

[B18] Shendure J Ji H Next-generation DNA sequencing Nat Biotechnol 2008 26 10 1135 1145 10.1038/nbt1486 18846087

[B19] Droege M Hill B The Genome Sequencer FLX System–longer reads, more applications, straight forward bioinformatics and more complete data sets J Biotechnol 2008 136 1–2 3 10 18616967 10.1016/j.jbiotec.2008.03.021

[B20] Kumar S Blaxter ML Comparing de novo assemblers for 454 transcriptome data BMC genomics 2010 11 571 10.1186/1471-2164-11-571 20950480 PMC3091720

[B21] Channeliere S Riviere S Scalliet G Szecsi J Jullien F Dolle C Vergne P Dumas C Bendahmane M Hugueney P Analysis of gene expression in rose petals using expressed sequence tags FEBS Lett 2002 515 1–3 35 38 11943190 10.1016/s0014-5793(02)02413-4

[B22] Jones-Rhoades MW Bartel DP Bartel B MicroRNAS and their regulatory roles in plants Annu Rev Plant Biol 2006 57 19 53 10.1146/annurev.arplant.57.032905.105218 16669754

[B23] Voinnet O Origin, biogenesis, and activity of plant microRNAs Cell 2009 136 4 669 687 10.1016/j.cell.2009.01.046 19239888

[B24] Friedlander MR Chen W Adamidi C Maaskola J Einspanier R Knespel S Rajewsky N Discovering microRNAs from deep sequencing data using miRDeep Nat Biotechnol 2008 26 4 407 415 10.1038/nbt1394 18392026

[B25] Zhu E Zhao F Xu G Hou H Zhou L Li X Sun Z Wu J mirTools: microRNA profiling and discovery based on high-throughput sequencing Nucleic Acids Res 2010 38 Web Server issue W392 W397 20478827 10.1093/nar/gkq393 PMC2896132

[B26] Hackenberg M Rodriguez-Ezpeleta N Aransay AM miRanalyzer: an update on the detection and analysis of microRNAs in high-throughput sequencing experiments Nucleic Acids Res 2011 39 Web Server issue W132 W138 21515631 10.1093/nar/gkr247 PMC3125730

[B27] Wang WC Lin FM Chang WC Lin KY Huang HD Lin NS miRExpress: analyzing high-throughput sequencing data for profiling microRNA expression BMC Bioinforma 2009 10 328 10.1186/1471-2105-10-328 PMC2767369 19821977

[B28] Yang X Li L miRDeep-P: a computational tool for analyzing the microRNA transcriptome in plants Bioinformatics 2011 27 18 2614 2615 21775303 10.1093/bioinformatics/btr430

[B29] Thakur V Wanchana S Xu M Bruskiewich R Quick WP Mosig A Zhu XG Characterization of statistical features for plant microRNA prediction BMC genomics 2011 12 108 10.1186/1471-2164-12-108 21324149 PMC3053258

[B30] Griffiths-Jones S Saini HK van Dongen S Enright AJ miRBase: tools for microRNA genomics Nucleic Acids Res 2008 36 Database issue D154 158 17991681 10.1093/nar/gkm952 PMC2238936

[B31] Zhang Z Yu J Li D Liu F Zhou X Wang T Ling Y Su Z PMRD: plant microRNA database Nucleic Acids Res 2010 38 Database issue D806 81 19808935 10.1093/nar/gkp818 PMC2808885

[B32] Huang X Madan A CAP3: A DNA sequence assembly program Genome Res 1999 9 9 868 877 10.1101/gr.9.9.868 10508846 PMC310812

[B33] Jaillon O Aury JM Noel B Policriti A Clepet C Casagrande A Choisne N Aubourg S Vitulo N Jubin C The grapevine genome sequence suggests ancestral hexaploidization in major angiosperm phyla Nature 2007 449 7161 463 467 10.1038/nature06148 17721507

[B34] Li L Stoeckert CJ Jr Roos DS OrthoMCL: identification of ortholog groups for eukaryotic genomes Genome Res 2003 13 9 2178 2189 10.1101/gr.1224503 12952885 PMC403725

[B35] Lamesch P Berardini TZ Li D Swarbreck D Wilks C Sasidharan R Muller R Dreher K Alexander DL Garcia-Hernandez M The Arabidopsis Information Resource (TAIR): improved gene annotation and new tools Nucleic Acids Res 2012 40 Database issue D1202 1210 22140109 10.1093/nar/gkr1090 PMC3245047

[B36] Xie Z Johansen LK Gustafson AM Kasschau KD Lellis AD Zilberman D Jacobsen SE Carrington JC Genetic and functional diversification of small RNA pathways in plants PLoS Biol 2004 2 5 E104 10.1371/journal.pbio.0020104 15024409 PMC350667

[B37] Breakfield NW Corcoran DL Petricka JJ Shen J Sae-Seaw J Rubio-Somoza I Weigel D Ohler U Benfey PN High-resolution experimental and computational profiling of tissue-specific known and novel miRNAs in Arabidopsis Genome Res 2012 22 1 163 176 10.1101/gr.123547.111 21940835 PMC3246203

[B38] Bolduc F Hoareau C St-Pierre P Perreault JP In-depth sequencing of the siRNAs associated with peach latent mosaic viroid infection BMC Mol Biol 2010 11 16 10.1186/1471-2199-11-16 20158907 PMC2830927

[B39] Meyers BC Axtell MJ Bartel B Bartel DP Baulcombe D Bowman JL Cao X Carrington JC Chen X Green PJ Criteria for annotation of plant MicroRNAs Plant Cell 2008 20 12 3186 3190 10.1105/tpc.108.064311 19074682 PMC2630443

[B40] Mi S Cai T Hu Y Chen Y Hodges E Ni F Wu L Li S Zhou H Long C Sorting of small RNAs into Arabidopsis argonaute complexes is directed by the 5' terminal nucleotide Cell 2008 133 1 116 127 10.1016/j.cell.2008.02.034 18342361 PMC2981139

[B41] Kim VN Sorting out small RNAs Cell 2008 133 1 25 26 10.1016/j.cell.2008.03.015 18394983

[B42] Llave C Xie Z Kasschau KD Carrington JC Cleavage of Scarecrow-like mRNA targets directed by a class of Arabidopsis miRNA Science 2002 297 5589 2053 2056 10.1126/science.1076311 12242443

[B43] Jones-Rhoades MW Bartel DP Computational identification of plant MicroRNAs and their targets, including a stress-induced miRNA Molecular Cell 2004 14 6 787 799 10.1016/j.molcel.2004.05.027 15200956

[B44] Hatlestad GJ Sunnadeniya RM Akhavan NA Gonzalez A Goldman IL McGrath JM Lloyd AM The beet R locus encodes a new cytochrome P450 required for red betalain production Nat Genet 2012 44 7 816 820 10.1038/ng.2297 22660548

[B45] Tanaka Y Flower colour and cytochromes P450 Phytochem Rev 2006 5 583 291

[B46] Dubos C Stracke R Grotewold E Weisshaar B Martin C Lepiniec L MYB transcription factors in Arabidopsis Trends Plant Sci 2010 15 10 573 581 10.1016/j.tplants.2010.06.005 20674465

[B47] Gou JY Felippes FF Liu CJ Weigel D Wang JW Negative regulation of anthocyanin biosynthesis in Arabidopsis by a miR156-targeted SPL transcription factor Plant Cell 2011 23 4 1512 1522 10.1105/tpc.111.084525 21487097 PMC3101539

[B48] Moxon S Jing R Szittya G Schwach F Rusholme Pilcher RL Moulton V Dalmay T Deep sequencing of tomato short RNAs identifies microRNAs targeting genes involved in fruit ripening Genome Res 2008 18 10 1602 1609 10.1101/gr.080127.108 18653800 PMC2556272

[B49] Fahlgren N Howell MD Kasschau KD Chapman EJ Sullivan CM Cumbie JS Givan SA Law TF Grant SR Dangl JL High-throughput sequencing of Arabidopsis microRNAs: evidence for frequent birth and death of MIRNA genes PLoS One 2007 2 2 e219 10.1371/journal.pone.0000219 17299599 PMC1790633

[B50] Fahlgren N Jogdeo S Kasschau KD Sullivan CM Chapman EJ Laubinger S Smith LM Dasenko M Givan SA Weigel D MicroRNA gene evolution in Arabidopsis lyrata and Arabidopsis thaliana The Plant cell 2010 22 4 1074 1089 10.1105/tpc.110.073999 20407027 PMC2879733

[B51] Pantaleo V Szittya G Moxon S Miozzi L Moulton V Dalmay T Burgyan J Identification of grapevine microRNAs and their targets using high-throughput sequencing and degradome analysis The Plant journal: for cell and molecular biology 2010 62 6 960 976 20230504 10.1111/j.0960-7412.2010.04208.x

[B52] Yang X Zhang H Li L Global analysis of gene-level microRNA expression in Arabidopsis using deep sequencing data Genomics 2011 98 1 40 46 21473907 10.1016/j.ygeno.2011.03.011

[B53] Wang C Wang X Kibet NK Song C Zhang C Li X Han J Fang J Deep sequencing of grapevine flower and berry short RNA library for discovery of novel microRNAs and validation of precise sequences of grapevine microRNAs deposited in miRBase Physiol Plant 2011 143 1 64 81 10.1111/j.1399-3054.2011.01481.x 21496033

[B54] Wei LQ Yan LF Wang T Deep sequencing on genome-wide scale reveals the unique composition and expression patterns of microRNAs in developing pollen of Oryza sativa Genome Biol 2011 12 6 R53 10.1186/gb-2011-12-6-r53 21679406 PMC3218841

[B55] Barakat A Wall K Leebens-Mack J Wang YJ Carlson JE Depamphilis CW Large-scale identification of microRNAs from a basal eudicot (Eschscholzia californica) and conservation in flowering plants The Plant journal: for cell and molecular biology 2007 51 6 991 1003 10.1111/j.1365-313X.2007.03197.x 17635767

[B56] Li H Zhang Z Huang F Chang L Ma Y MicroRNA expression profiles in conventional and micropropagated strawberry (Fragaria x ananassa Duch.) plants Plant cell reports 2009 28 6 891 902 10.1007/s00299-009-0693-3 19277667

[B57] Cuperus JT Fahlgren N Carrington JC Evolution and functional diversification of MIRNA genes Plant Cell 2011 23 2 431 442 10.1105/tpc.110.082784 21317375 PMC3077775

[B58] Xu Q Liu Y Zhu A Wu X Ye J Yu K Guo W Deng X Discovery and comparative profiling of microRNAs in a sweet orange red-flesh mutant and its wild type BMC genomics 2010 11 246 10.1186/1471-2164-11-246 20398412 PMC2864249

[B59] Donaire L Pedrola L Rosa Rde L Llave C High-throughput sequencing of RNA silencing-associated small RNAs in olive (Olea europaea L) PLoS One 2011 6 11 e27916 10.1371/journal.pone.0027916 22140484 PMC3225373

[B60] Achard P Herr A Baulcombe DC Harberd NP Modulation of floral development by a gibberellin-regulated microRNA Development 2004 131 14 3357 3365 10.1242/dev.01206 15226253

[B61] Schwab R Palatnik JF Riester M Schommer C Schmid M Weigel D Specific effects of microRNAs on the plant transcriptome Developmental cell 2005 8 4 517 527 10.1016/j.devcel.2005.01.018 15809034

[B62] Wu MF Tian Q Reed JW Arabidopsis microRNA167 controls patterns of ARF6 and ARF8 expression, and regulates both female and male reproduction Development 2006 133 21 4211 4218 10.1242/dev.02602 17021043

[B63] Chen X A microRNA as a translational repressor of APETALA2 in Arabidopsis flower development Science 2004 303 5666 2022 2025 10.1126/science.1088060 12893888 PMC5127708

[B64] Aukerman MJ Sakai H Regulation of flowering time and floral organ identity by a MicroRNA and its APETALA2-like target genes Plant Cell 2003 15 11 2730 2741 10.1105/tpc.016238 14555699 PMC280575

[B65] Mallory AC Bartel DP Bartel B MicroRNA-directed regulation of Arabidopsis AUXIN RESPONSE FACTOR17 is essential for proper development and modulates expression of early auxin response genes Plant Cell 2005 17 5 1360 1375 10.1105/tpc.105.031716 15829600 PMC1091760

[B66] Wang JW Wang LJ Mao YB Cai WJ Xue HW Chen XY Control of root cap formation by MicroRNA-targeted auxin response factors in Arabidopsis Plant Cell 2005 17 8 2204 2216 10.1105/tpc.105.033076 16006581 PMC1182483

[B67] Garcia D A miRacle in plant development: role of microRNAs in cell differentiation and patterning Seminars in cell & developmental biology 2008 19 6 586 595 10.1016/j.semcdb.2008.07.013 18708151

[B68] Nishihara M Nakatsuka T Genetic engineering of flavonoid pigments to modify flower color in floricultural plants Biotechnol Lett 2011 33 3 433 441 10.1007/s10529-010-0461-z 21053046

[B69] Tanaka Y Sasaki N Ohmiya A Biosynthesis of plant pigments: anthocyanins, betalains and carotenoids The Plant journal: for cell and molecular biology 2008 54 4 733 749 10.1111/j.1365-313X.2008.03447.x 18476875

[B70] Chiou CY Yeh KW Differential expression of MYB gene (OgMYB1) determines color patterning in floral tissue of Oncidium Gower Ramsey Plant Mol Biol 2008 66 4 379 388 10.1007/s11103-007-9275-3 18161007

[B71] Lin-Wang K Bolitho K Grafton K Kortstee A Karunairetnam S McGhie TK Espley RV Hellens RP Allan AC An R2R3 MYB transcription factor associated with regulation of the anthocyanin biosynthetic pathway in Rosaceae BMC plant biology 2010 10 50 10.1186/1471-2229-10-50 20302676 PMC2923524

[B72] Xia R Zhu H An YQ Beers EP Liu Z Apple miRNAs and tasiRNAs with novel regulatory networks Genome Biol 2012 13 6 R47 10.1186/gb-2012-13-6-r47 22704043 PMC3446319

[B73] Suzuki Y Mae T Makino A RNA extraction from various recalcitrant plant tissues with a cethyltrimethylammonium bromide-containing buffer followed by an acid guanidium thiocyanate-phenol-chloroform treatment Biosci Biotechnol Biochem 2008 72 7 1951 1953 10.1271/bbb.80084 18603812

[B74] Lu C Meyers BC Green PJ Construction of small RNA cDNA libraries for deep sequencing Methods 2007 43 2 110 117 10.1016/j.ymeth.2007.05.002 17889797

[B75] Pruitt KD Tatusova T Klimke W Maglott DR NCBI Reference Sequences: current status, policy and new initiatives Nucleic Acids Res 2009 37 Database issue D32 36 18927115 10.1093/nar/gkn721 PMC2686572

[B76] Lamesch P Dreher K Swarbreck D Sasidharan R Reiser L Huala E Using the Arabidopsis information resource (TAIR) to find information about Arabidopsis genes Curr Protoc Bioinformatics 2010 Chapter 1 Unit1 11 20521243 10.1002/0471250953.bi0111s30

[B77] Aoki KF Kanehisa M Using the KEGG database resource Curr Protoc Bioinformatics 2005 Chapter 1 Unit 1 12 18428742 10.1002/0471250953.bi0112s11

[B78] Ruepp A Zollner A Maier D Albermann K Hani J Mokrejs M Tetko I Guldener U Mannhaupt G Munsterkotter M The FunCat, a functional annotation scheme for systematic classification of proteins from whole genomes Nucleic Acids Res 2004 32 18 5539 5545 10.1093/nar/gkh894 15486203 PMC524302

[B79] Jaiswal P Avraham S Ilic K Kellogg EA McCouch S Pujar A Reiser L Rhee SY Sachs MM Schaeffer M Plant Ontology (PO): a Controlled Vocabulary of Plant Structures and Growth Stages Comparative and functional genomics 2005 6 7–8 388 397 18629207 10.1002/cfg.496 PMC2447502

[B80] Avraham S Tung CW Ilic K Jaiswal P Kellogg EA McCouch S Pujar A Reiser L Rhee SY Sachs MM The Plant Ontology Database: a community resource for plant structure and developmental stages controlled vocabulary and annotations Nucleic Acids Res 2008 36 Database issue) D449 454 18194960 10.1093/nar/gkm908 PMC2238838

[B81] Mulder N Apweiler R InterPro and InterProScan: tools for protein sequence classification and comparison Methods Mol Biol 2007 396 59 70 10.1007/978-1-59745-515-2_5 18025686

[B82] Blankenberg D Gordon A Von Kuster G Coraor N Taylor J Nekrutenko A Manipulation of FASTQ data with Galaxy Bioinformatics 2010 26 14 1783 1785 10.1093/bioinformatics/btq281 20562416 PMC2894519

[B83] Gardner PP Daub J Tate J Moore BL Osuch IH Griffiths-Jones S Finn RD Nawrocki EP Kolbe DL Eddy SR Rfam: Wikipedia, clans and the "decimal" release Nucleic Acids Res 2011 39 Database issue D141 145 21062808 10.1093/nar/gkq1129 PMC3013711

[B84] Brown JW Echeverria M Qu LH Lowe TM Bachellerie JP Huttenhofer A Kastenmayer JP Green PJ Shaw P Marshall DF Plant snoRNA database Nucleic Acids Res 2003 31 1 432 435 10.1093/nar/gkg009 12520043 PMC165456

[B85] Schattner P Brooks AN Lowe TM The tRNAscan-SE, snoscan and snoGPS web servers for the detection of tRNAs and snoRNAs Nucleic Acids Res 2005 33 Web Server issue W686 689 15980563 10.1093/nar/gki366 PMC1160127

[B86] Kozomara A Griffiths-Jones S miRBase: integrating microRNA annotation and deep-sequencing data Nucleic Acids Res 2011 39 Database issue D152 157 21037258 10.1093/nar/gkq1027 PMC3013655

[B87] Dai X Zhao PX psRNATarget: a plant small RNA target analysis server Nucleic Acids Res 2011 39 Web Server issue W155 W159 21622958 10.1093/nar/gkr319 PMC3125753

[B88] Audic S Claverie JM The significance of digital gene expression profiles Genome Res 1997 7 10 986 995 9331369 10.1101/gr.7.10.986

