# Supplementary figures and images for: Small RNA and transcriptome deep sequencing proffers insight into floral gene regulation in Rosa cultivars
Source: BMC Genomics. 2012 Nov 21;13:657. doi: 10.1186/1471-2164-13-657 (PMC3527192; doi:10.1186/1471-2164-13-657)

Additional File 1. Small RNA length Distribution (raw data)

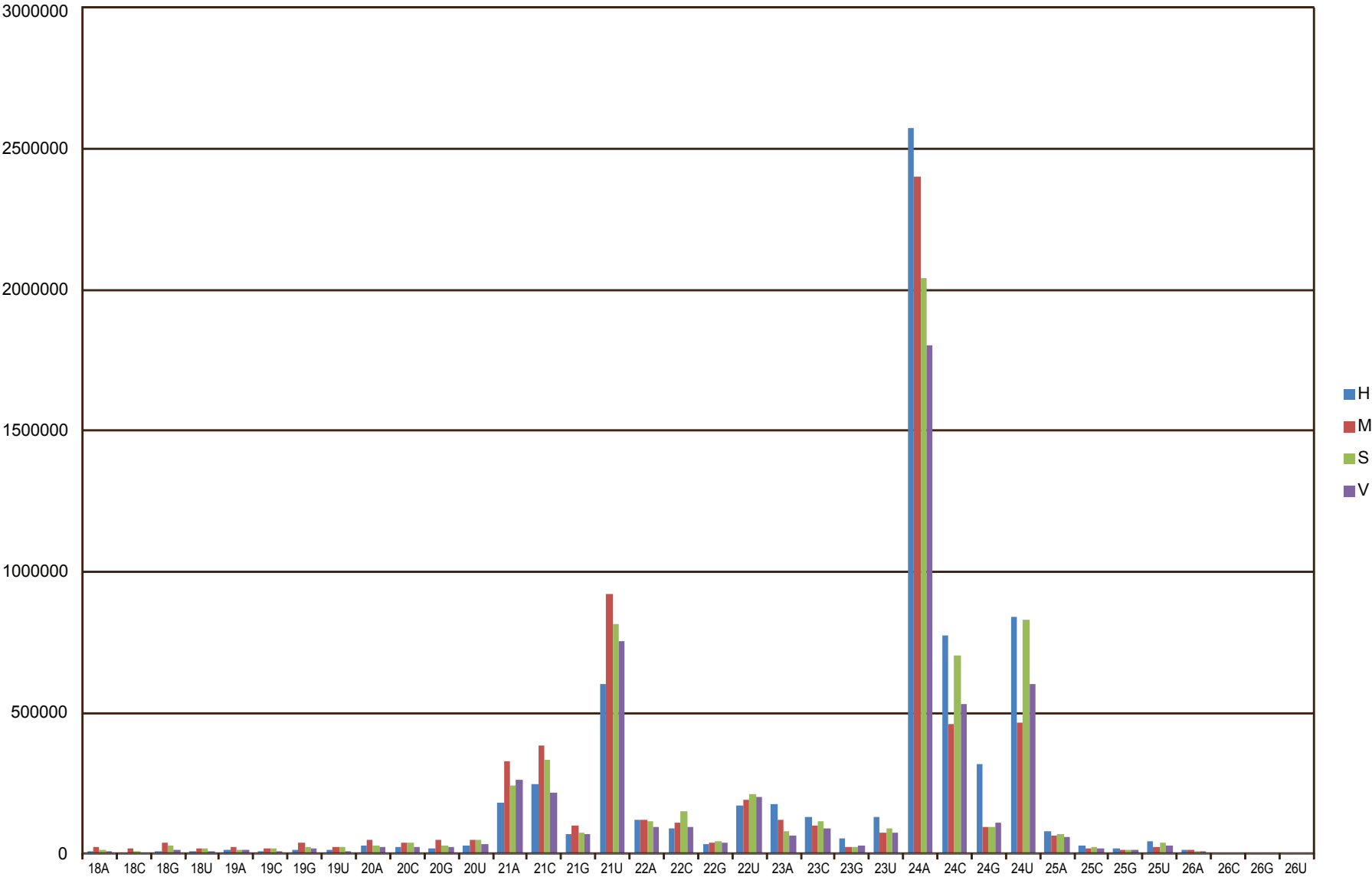

Supplement: Additional file 1 — The statistics of sRNA and its distribution. [file 1471-2164-13-657-S1.pdf]

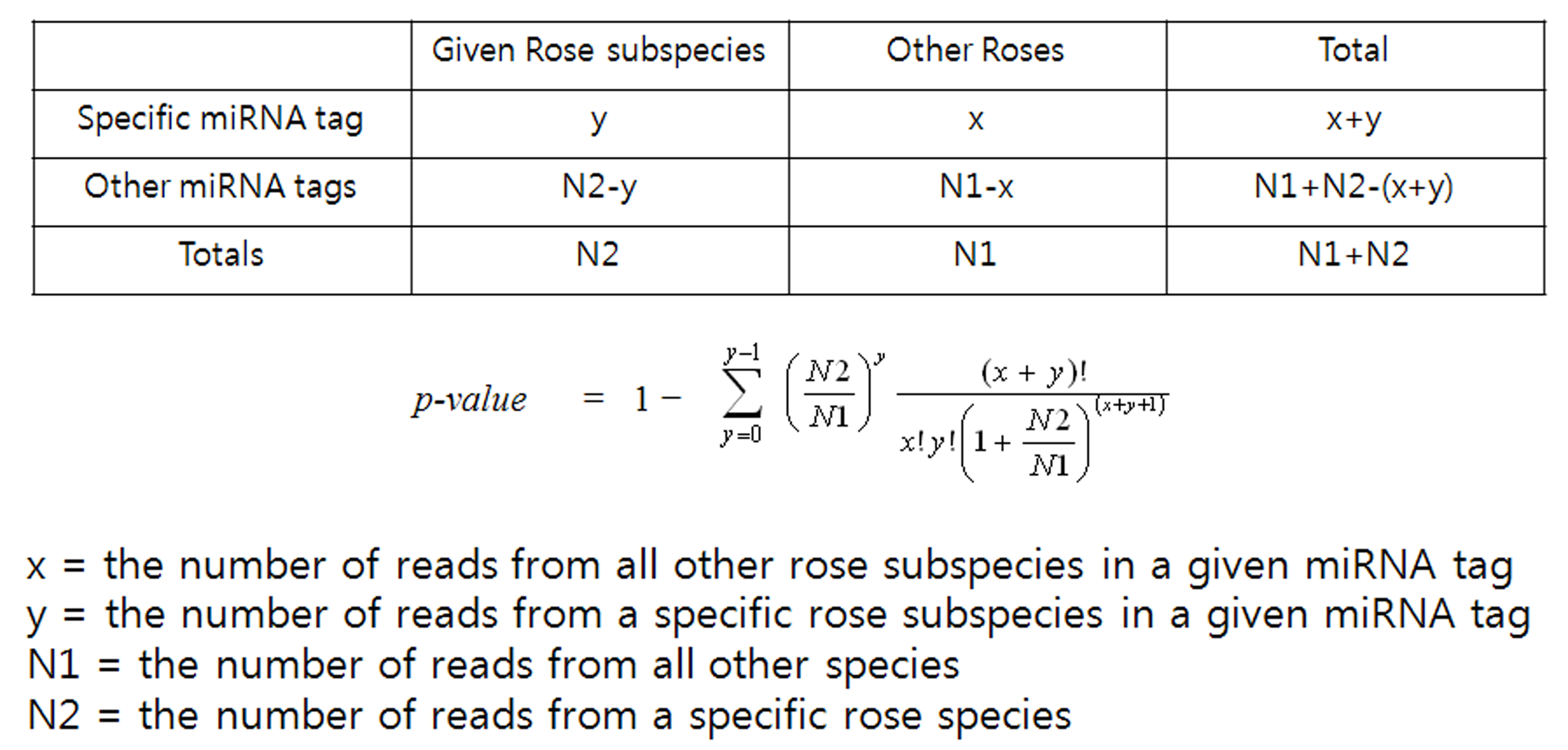

Supplement: Additional file 8 — The 2 * 2 contingency table and the probability equation for Audic’s test. [file 1471-2164-13-657-S8.jpeg]
