# Supplementary material for: Small RNA and transcriptome deep sequencing proffers insight into floral gene regulation in Rosa cultivars
Source: BMC Genomics. 2012 Nov 21;13:657. doi: 10.1186/1471-2164-13-657 (PMC3527192; doi:10.1186/1471-2164-13-657)
Supplement: Additional file 5 — Schematic diagram of flavonoid biosynthetic pathways and miRNA profiles targeting flavonoid biosynthetic genes. Colours represent enrichment genes in specific Rosa measured by applying Audic’s test (p value < 0.001).* miRNA cleavage were experimentally validated (Figure 5). [file 1471-2164-13-657-S5.pptx]

## Slide 1
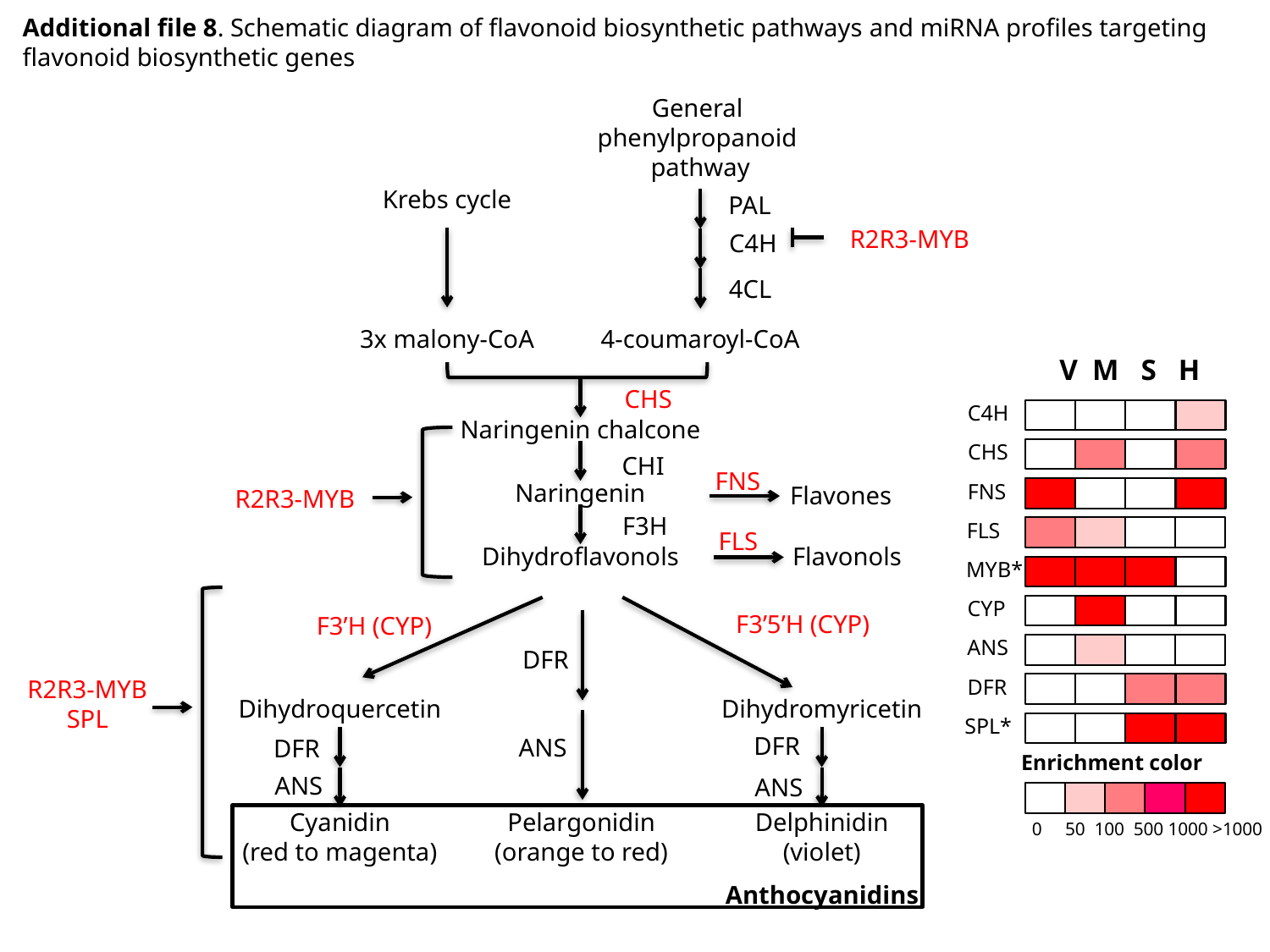

Additional file 8. Schematic diagram of flavonoid biosynthetic pathways and miRNA profiles targeting flavonoid biosynthetic genes
General
phenylpropanoid
pathway
PAL
C4H
4CL
4-coumaroyl-CoA
Krebs cycle
3x malony-CoA
Naringenin chalcone
Naringenin
Dihydroflavonols
CHS
CHI
F3H
Dihydroquercetin
Cyanidin
(red to magenta)
Dihydromyricetin
Delphinidin
(violet)
Pelargonidin
(orange to red)
F3’5’H (CYP)
F3’H (CYP)
DFR
DFR
ANS
DFR
ANS
ANS
R2R3-MYB
R2R3-MYB
R2R3-MYB
SPL
Anthocyanidins
FNS
Flavones
FLS
Flavonols
V M S H
C4H
CHS
FNS
FLS
MYB*
CYP
ANS
DFR
SPL*
Enrichment color
0 50 100 500 1000 >1000
